# Is High Temporal Resolution Achievable for Paediatric Cardiac Acquisitions during Several Heart Beats? Illustration with Cardiac Phase Contrast Cine-MRI

**DOI:** 10.1371/journal.pone.0143744

**Published:** 2015-11-24

**Authors:** Laurent Bonnemains, Freddy Odille, Christophe Meyer, Gabriella Hossu, Jacques Felblinger, Pierre-André Vuissoz

**Affiliations:** 1 Department of Cardiology, CHU Strasbourg, Strasbourg, France; 2 Department of Cardiology, CHU Nancy, Nancy, France; 3 U947, INSERM, Nancy, France; 4 IADI, University of Lorraine, Nancy, France; 5 Clinical Investigation Center (CIC-IT 1433), CHU Nancy, Nancy, France; University of Washington, UNITED STATES

## Abstract

**Background:**

During paediatric cardiac Cine-MRI, data acquired during cycles of different lengths must be combined. Most of the time, Feinstein’s model is used to project multiple cardiac cycles of variable lengths into a mean cycle.

**Objective:**

To assess the effect of Feinstein projection on temporal resolution of Cine-MRI.

**Methods:**

1/The temporal errors during Feinstein’s projection were computed in 306 cardiac cycles fully characterized by tissue Doppler imaging with 6-phase analysis (from a population of 7 children and young adults). 2/The effects of these temporal errors on tissue velocities were assessed by simulating typical tissue phase mapping acquisitions and reconstructions. 3/Myocardial velocities curves, extracted from high-resolution phase-contrast cine images, were compared for the 6 volunteers with lowest and highest heart rate variability, within a population of 36 young adults.

**Results:**

1/The mean of temporal misalignments was 30 ms over the cardiac cycle but reached 60 ms during early diastole. 2/During phase contrast MRI simulation, early diastole velocity peaks were diminished by 6.1 cm/s leading to virtual disappearance of isovolumic relaxation peaks. 3/The smoothing and erasing of isovolumic relaxation peaks was confirmed on tissue phase mapping velocity curves, between subjects with low and high heart rate variability (p = 0.05).

**Conclusions:**

Feinstein cardiac model creates temporal misalignments that impair high temporal resolution phase contrast cine imaging when beat-to-beat heart rate is changing.

## Introduction

**Fig 1:**
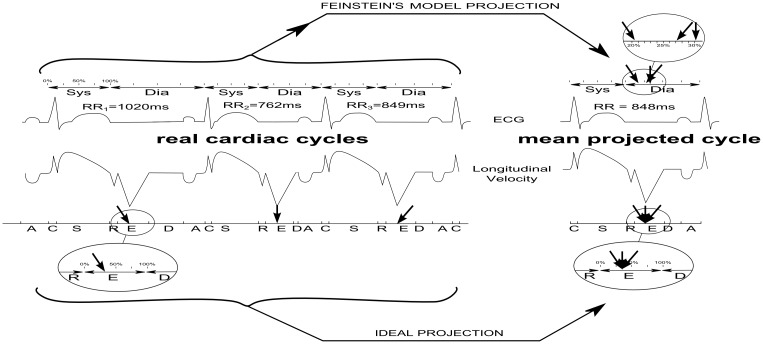
Illustration of the reconstruction process consisting in projecting several cardiac cycles with beat-to-beat variations into a mean cardiac cycle. Left side of the Figure represent three example cardiac cycles (left top is ECG and left bottom is TDI displacement). Below TDI curve are letters corresponding to cardiac phases (C = isovolumic Contraction, S = Systolic ejection, R = isovolumic Relaxation, E = Early diastole filling, D = Diastasis, and A = Atrial contraction). Right side has a top part (2 phase model) showing how the peak E wave positions do not temporally align, whereas bottom part (6 phase model) shows the alignment much better. The two circles represent zoomed part of the TDI displacement curves.

Imaging the beating heart may require to span the acquisition over several heart beats when the imaging modality is too slow. It is mainly the case of cine-MRI but also of 3D “non-live” echocardiography. This is conventionally achieved with a segmented acquisition strategy: the acquisition space (k-space for MRI) is divided into several segments and a given segment is repeatedly acquired during a complete cardiac cycle. Therefore, a n-segment acquisition is spanned over n cardiac cycles. This strategy requires a reconstruction process to recombine segments acquired during different cardiac cycles in order to form complete datasets. In the field of cardiac MRI, several reconstruction strategies are currently available: 1/The trivial prospective acquisition/reconstruction (originating from the early developments of cardiac MRI) consists in grouping all segments acquired at the same delay to the R-wave (called trigger delay) in order to form a given image of the cine movie. However since the heart rate varies from beat to beat, data from the end of the longer cardiac cycles have to be discarded, leading to suboptimal reconstruction of the cardiac cycle. 2/To solve this problem Feinstein *et al*. proposed to split the cardiac cycle into 2 phases (i.e. systole and diastole) and to retrospectively apply a linear stretching to these two phases [1], as illustrated in Fig 1 (upper part of the Figure). This linear stretching projects the acquired segments at different moments of the cardiac cycle. The latter strategy ensures that data from the whole cardiac cycle are effectively used at the reconstruction stage. However this strategy relies on two assumptions. The first hypothesis is that reconstruction based on a two-phase cardiac model is efficient. The second hypothesis is that the duration of systole and diastole can be predicted for any individual patient by the formula proposed by Weissler *et al*. [2] Weissler’s formula was derived by linear regression from the mean of 20 to 30 consecutive measurements of systole and RR durations in a population of 221 healthy volunteers using a 5ms temporal resolution method.

These two hypotheses (the two-phase cardiac model and Weissler’s population-based formula) obviously constitute two different approximations that may result in temporal misalignments between k-space lines in cases of heart beat to beat variations, as illustrated in Fig 1. As a consequence, segments corresponding to different heart positions could be wrongly combined during reconstruction. To our knowledge the influence of these two hypotheses on cine MR temporal resolution has not been investigated. These temporal misalignments in the k-space could impair the detection of very short phenomenon and in that way alter the temporal resolution. This may probably be neglected whenever these misalignments are smaller than the desired temporal resolution, which is the case for usual cine-MRI acquisition. Indeed, typical parameters for cardiac function assessment with a cine MRI sequence are: Time of Repetition (TR) close to 4ms, and number of k-space lines contained in each segment, called views per segment (VPS) close to 12, and, consequently the temporal resolution of such typical acquisitions is close to 50ms. However the impact of those temporal misalignments should be studied and discussed whenever high temporal resolution (and a high number of images per cycle) is used as can be found in several recent publications [3–9].

The objective of this study was to prove that these temporal misalignments impair cine MRI. Firstly, we quantified the errors induced by the Feinstein model using real-time tissue Doppler imaging (TDI) data acquired over multiple heart beats in children with high heart rate variability. Secondly, we assessed the impact of such errors during phase contrast cine MRI with a MRI simulator. And thirdly, we verified the results of the simulation with a real high temporal resolution phase-contrast cine MRI experiment including normal subjects with either low or high heart rate variability.

## Methods

### Real-time tissue Doppler imaging

306 cardiac cycles were acquired from 7 children or young adults aged from 2 to 20 (mean = 11+/-7y). This population was chosen because of the higher influence of vagal tonus at this age [10]. Data were collected during conventional echocardiographies planned to monitor cardiac function. The examinations were scheduled more than one year after the chemotherapy treatment of a leukaemia or solid cancer. The patients’ characteristics are summarized in Table 1.

**Table 1:** Patients Characteristics (used to collect data for the simulation part).

Patient	Age(y)	Sex M/F	Weight(kg)	Height(cm)	Heart beat(bpm)	SD_RR_ (ms)	Cardiac cycles^a^
1	13	M	42	150	71	53	30
2	3	M	14	95	92	98	21
3	20	M	67	169	91	30	44
4	9	F	33	133	80	62	55
5	14	F	48	157	73	155	45
6	15	M	65	175	89	23	52
7	2	M	14	102	102	52	59
							Total = 306
Mean(SD)	11 (7)		40 (22)	140 (32)	86 (11)	68 (45)	44 (14)

Legend: M/F = Male, Female; SD_RR_ = standard deviation of cardiac cycle duration

^a^longer span of consecutive cardiac cycles with sufficient quality to be manually annotated

#### Ethics statements

This part of the study is a non-interventional study, as defined by European law (EU directive on clinical trials 2001/20/EC): 1/no medicinal product; 2/no randomisation; 3/no additional diagnostic or monitoring procedure. Non-interventional or retrospective studies do not require written patient consent. However, in our case, patients and/or their parents signed a written non-opposition form (approved by a local ethical committee) and additionally also gave oral informed consent (mentioned in the source documents of the patient). This part of the study was registered under the id 1720841 by our national registration system CNIL. It complies with the Declaration of Helsinki concerning medical research on human subjects and to national and European laws.

**Fig 2:**
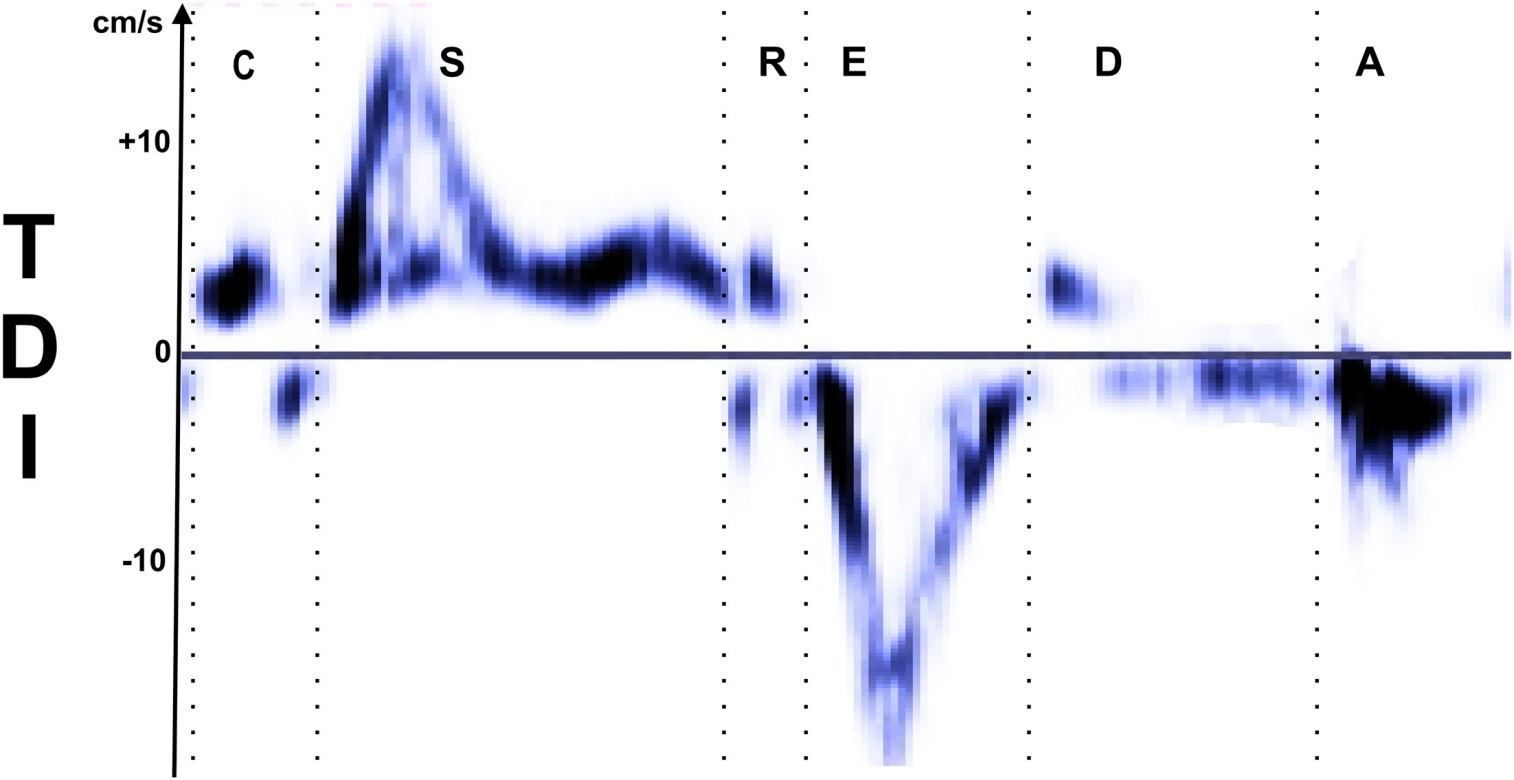
Example of myocardial velocity curves in the longitudinal axis and definitions of the six cardiac phases (C, S, R, E, D, and A) used in this study. C is considered as the beginning of the systole and of the cycle. A is considered as the last part of the diastole and of the cycle.

All patient echocardiography were judged normal (Left Ventricle Ejection Fraction ≥ 60% and Myocardial Performance Index < 0.40). Echocardiographies were performed using a Vivid 7 Ultrasound system (General Electric Vingmed Ultrasound, Horten, Norway) by an experienced cardiologist. Patients were in a quiet state without sedation. TDI of the four-chamber (horizontal long axis) view was acquired with electrocardiogram recording. The orientation was chosen carefully to align the sample volume motion and the beam as much as possible, as recommended [11]. The Doppler sample volume was positioned on the lateral wall of the left ventricle at 1 cm of the mitral valve. It was adjusted as necessary to cover the longitudinal excursion of the annulus in both systole and diastole [11]. The velocity curve was recorded for about one minute (as presented in S1 File). Each cycle was then manually divided into 6 phases [11,12] by an experienced cardiologist: isovolumic contraction (C) peak, systolic (S) wave, isovolumic relaxation (R), early diastole (E) wave, diastasis (D), and auricular (A) wave. An example of the division of these TDI waves into 6 phases is presented in Fig 2. The duration of each phase was measured during 44+/-14 consecutive cardiac cycles (min = 21, max = 59). According to the literature in which the same hardware is employed [4,13] and to the user guide, the temporal resolution of the TDI scan should be higher than 200 frames/s. However, this information is not directly accessible on our ultrasound system.

### Quantification of the Feinstein cardiac model errors

These 306 cardiac cycles were used to assess the time misalignments created by Feinstein model during reconstruction. Each cardiac cycle was discretized into 1000 time steps (a ‘step’ corresponding to less than 1ms according to the heart rate). Each step of each cardiac cycle was projected onto a template cycle for each patient with a piecewise linear stretching using three different projection methods: 1/ Feinstein’s model that approximately realigns the bounds of systole and diastole, based on the estimated duration of systole and diastole from the instantaneous heart rate (with Weissler’s formula); 2/ the best possible two-phase model (hereafter called “best two-phase model”) that uses the manual annotations of end-systole (end of the S wave) from the TDI curves to perfectly realign the bounds of systole and diastole; 3/ the gold standard method for this study: model that uses all the annotations from the TDI curves to perfectly realigns the bounds of the six phases (C, S, R, E, D, A). Using the latter model as a gold-standard, the projection errors due to either the best two-phase model or Feinstein’s model were recorded for each of the 1000 steps. The limits of agreement were also computed for each step, as 1.96 times the standard deviation of these differences over the 306 cardiac cycles [14]. The global mean (for each phase and for the complete cardiac cycle) of these limits of agreement was also computed.

### Simulation of the resulting errors for a typical tissue phase contrast cine-MRI

**Fig 3:**
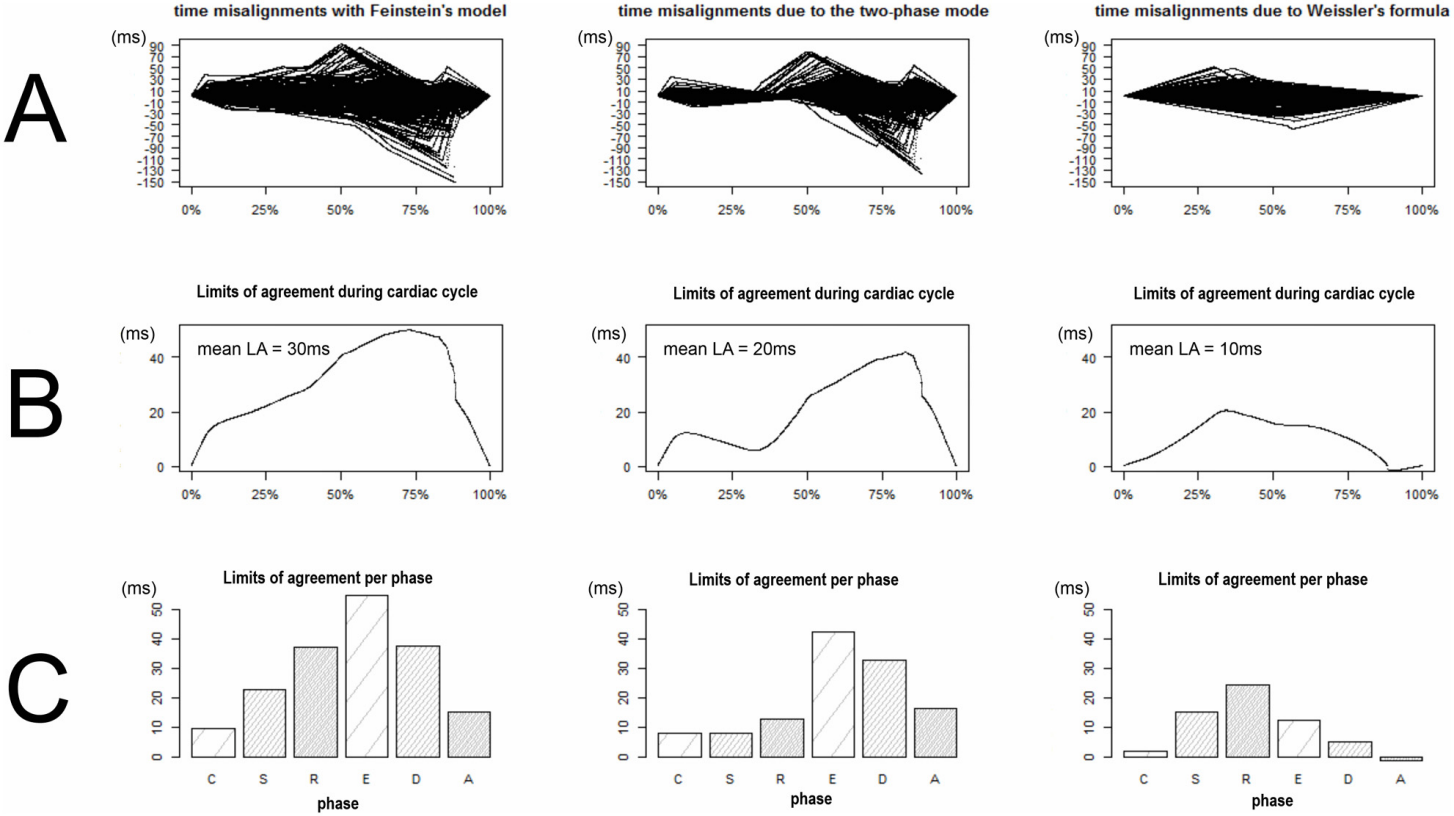
Tissue phase mapping MRI simulation with acquisition spanned over different cycles (patient 7). A template typical velocity curve (A) was used for the simulation. An acquisition spanned over cycles of the same duration results in the velocity curve (B). (C) represents the velocity curves obtained with varying filling order of the k-space. The dark line is the median curve and the +/-2SD space is represented in grey. (D) represents the velocity errors induced by the HR variability.

The consequences of the temporal misalignments induced by Feinstein’s model were highlighted by a simulation. A PC cine MRI simulator was implemented in the Julia programming language [15]. The MRI simulation consisted in three main steps:

1. Creation of a numerical velocity curve: For each patient, we generated a model of myocardial longitudinal velocity curve based on the 6-phase TDI annotations with triangular shapes for each cardiac phase (including sharp peaks of isovolumic contraction and isovolumic relaxation). The durations of each phase of the cardiac cycles were those measured by TDI echocardiography, and the value of the velocity was computed (using nearest neighbour interpolation) so that the velocity curve matched the template curve given in Fig 3(a).
2. Simulation of PC cine MRI acquisition: we generated simulated MRI raw data (i.e. k-space data) corresponding to a phase contrast acquisition. The acquisition parameters used by the simulator have been summarized in Table 2. The magnitude of the MR images was synthesized with the Shepp-Logan numerical 2D phantom (which is widely used in numerical simulations); the phase of the MRI images were calculated as if the whole phantom experienced a through-plane motion which velocity matched the previously constructed velocity curve. Fourier transform and k-line selection resulted in the simulated MRI raw data.
3. Cine MRI reconstruction: PC cine images (magnitude and velocity images) were then reconstructed from these raw data using Feinstein’s model. The mean velocity in the moving region was computed from the phase image and was used as output velocity curve.

**Table 2:** Characteristics of the MRI simulator.

MRI parameter used in simulator	Value
k-space and image matrix size	N^2^, N being the number of cardiac cycles
Velocity encoding (Venc)	Ideal (equation: max(abs(velocityTemplate)))
Repetition time (TR)	5 ms
Views per segment (VPS)	1
k-space acquisition order (viewtable)	Linear sequential
Number of velocity encoding steps	2
Velocity encoding mode	interleaved
Number of reconstructed cardiac phases	200
Reconstructed cardiac temporal window	10ms (= 2 TR)
Number of averages (NEX)	1
Number of coils	1
Number of slices	1

Steps 2 and 3 of this simulation were performed a second time with no heart rate variation to obtain a gold-standard reconstruction. For this purpose, the whole simulated acquisition was performed with the same cardiac cycle repeated and corresponding to the median cycle of the patient.

Because the order of the k-space filling (or the order of the cardiac cycles) could influence the results, we performed a Monte Carlo simulation: 100 MRI simulation/reconstruction experiments were performed, each experiment using a random selection of cardiac cycles from the numerical velocity curve.

### High-resolution PC cine MRI data

The above simulation was verified by comparing high temporal resolution tissue phase mapping myocardial velocity curves obtained from healthy volunteers with low and high cardiac cycle length (called RR: delay between two consecutive QRS) variability. To form these two groups of volunteers with high and low heart rate variability, we recruited 36 healthy volunteers and selected six of them: the “low RR variation” group comprised the three volunteers with the lowest RR standard deviation and the “high RR variation” group comprised the three volunteers with the highest RR standard deviation. The initial 36 volunteers were consecutive subjects from the whole cohort of the ESCIF program (prospective ancillary study of the STANISLAS project) [16]. The volunteers had a complete echocardiography examination just before the CMR examination and only those with normal echocardiography (notably normal TDI curves) had been included.

Ethics statements: This part of the study is prospective. It has been approved by a local ethical committee (“Comité de Protection des Personnes Est-III”–Hôpital de Brabois–Rue du Morvan–VANDOEUVRE LES NANCY Cedex, France) and complies with the Declaration of Helsinki concerning medical research on human subjects. The volunteers gave informed written consent.

The acquisitions were performed on a 3T MRI scanner (Signa HDx, General Electric, Waukesha, WI) with subjects in the supine position and using an eight-element cardiac phased-array coil. As previously described [4,17], PC cine images were acquired during free-breathing in a short-axis slice positioned at the base of the heart using a 2D segmented Fast Gradient Recalled Echo sequence with one-directional through-slice interleaved velocity encoding and the following parameters: 8 mm slice-thickness, 9.4 ms repetition time, 4.7 ms echo time, 15° flip angle, 1 View Per Segment, 3 number of excitations, field-of-view of 37.5 cm and a 256x128 acquisition matrix. These parameters correspond to a theoretical temporal resolution of 2*TR = 18.8ms. Reconstructions were performed using Feinstein’s model and the number of reconstructed images per cycle was fixed to 60. Velocities were measured with commercial dedicated software (FLOW 3.3 MR Flow Quantification Software, Medis medical imaging system, Leiden, Nederland). No image-based velocity offset correction technique was used.

### Statistics

Continuous variables were presented as mean+/-SD. The relation between the maximum time displacements and the maximum velocity errors were analyzed by Pearson correlation and linear regression. The heights of the isovolumic relaxation R peaks between the two groups (low and high RR variations) were compared by a unilateral non-parametric Mann-Whitney U test adapted for small groups [18]. p ≤ 0.05 was considered to be statistically significant. Temporal projection and statistics were performed with a dedicated software programmed in R 3.0.2013-5-12 (R Foundation for Statistical Computing, Vienna, Austria) [19].

## Results

### Quantification of Feinstein’s model errors

**Fig 4:**
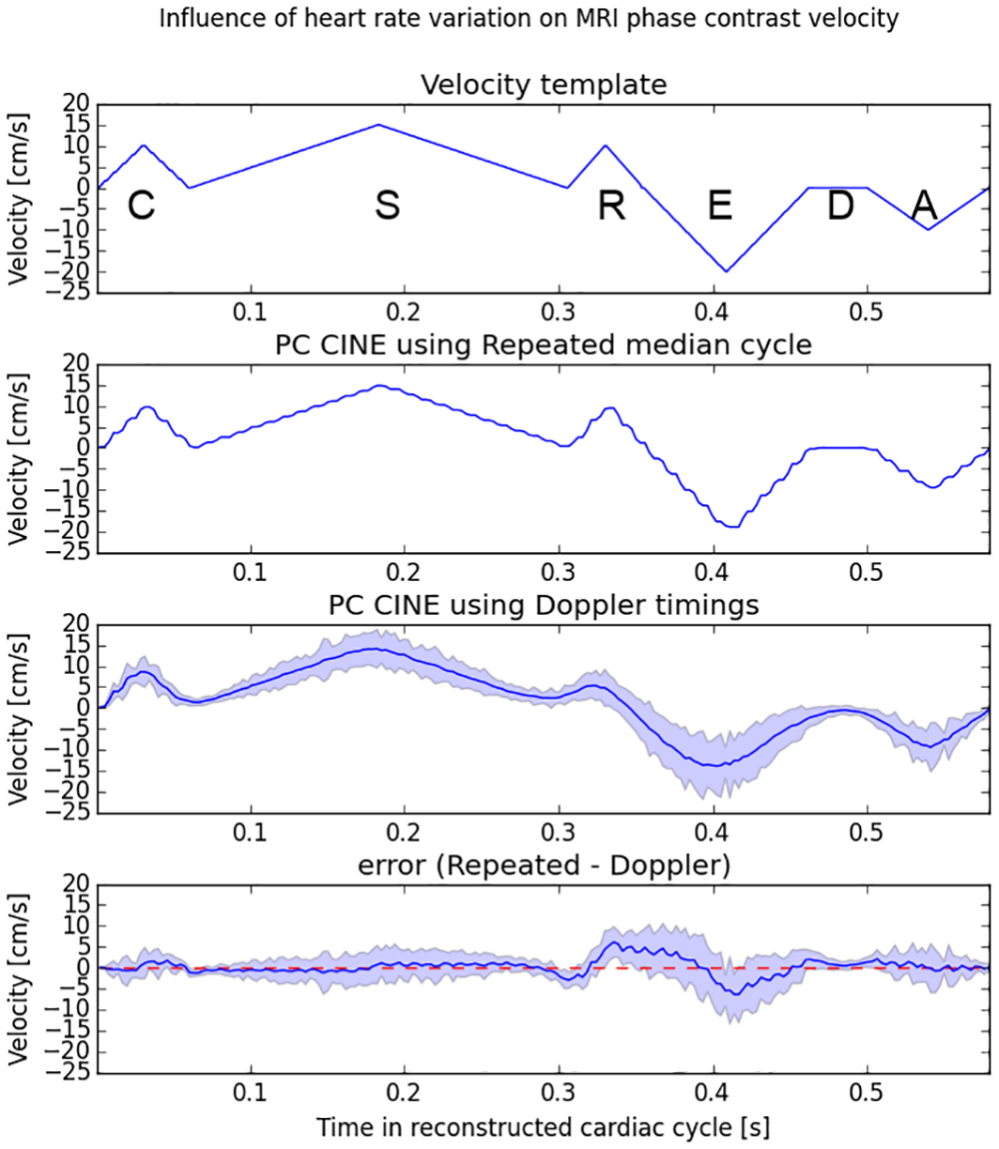
Time misalignments induced by Feinstein’s model (Left column) and their decomposition into what is caused by the use of a biphasic model (middle column) and by Weissler’s prediction formula (right column). (A) The top line plots represent the time misalignments for each position within the 306 cardiac cycle of the database (one black line per cardiac cycle). (B) The middle line plots represent the limit of agreement between each model and the ideal projection. (C) The bottom bar-plots represent the mean limit of agreement within each of the six phases.

The time shifts induced by Feinstein’s model and the best two-phase model were represented in Fig 4 (top line) as well as the limits of agreement per time step (second line) and per cardiac phase (third line). The difference between these time shifts were also computed and represented in the rightmost column of Fig 4 (This column represents what could be gained with a perfect prediction of the systole duration). The mean of the limits of agreement within the whole cardiac cycle was 30.2+/-1.6ms for Feinstein’s model and 20.1+/-1.1ms for the best two-phase model. However, during the start and middle of diastole, the time misalignments reached 60ms for Feinstein’s model and 40ms for the best two-phase model. During the early filling stage (E wave), the mean limit of agreement was 54.9+/-0.6ms with Feinstein’s model and 42.4+/-0.3ms with the best two-phase model. During the diastasis, the mean limit of agreement was 37.7+/-0.4ms with Feinstein’s model and 32.6+/-0.2ms with the best two-phase model.

### Phase contrast cine MRI simulation

**Fig 5:**
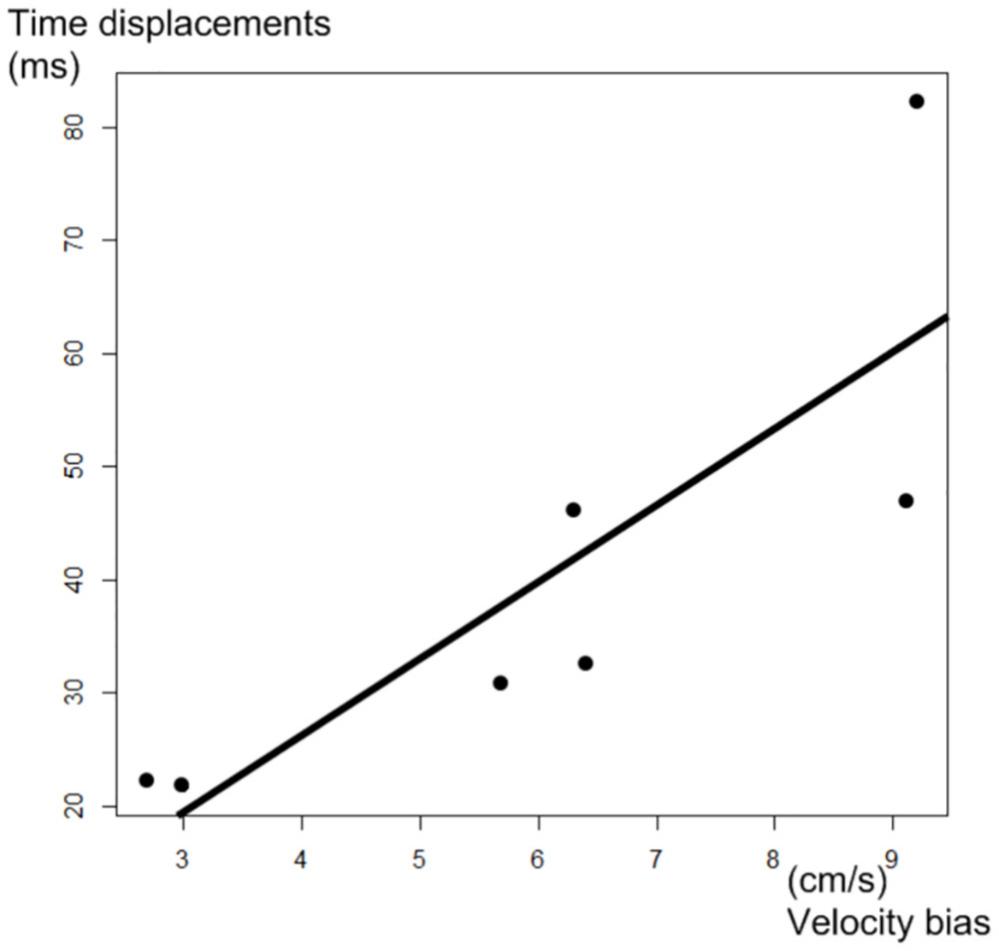
Influence of time displacements during Feinstein projection (y-axis) on velocity errors during phase-contrast acquisition (x-axis). Each point represents a distinct patient. The correlation is good (r^2^ = 0.7, p = 0.02) despite the small size of the sample.

Patient seven results are presented in Fig 3. The other patients’ results are provided in S1 Fig. Each figure comprises four curves: Curve a is the velocity template. Curve b is the computed velocity curve using the MRI simulator with the gold-standard projection method. Curve c is the reconstructed velocity curves using the model (Feinstein or best two-phase model). Mean and 1.96 x Standard deviation of the 100 reconstruction experiments are displayed. Curve d is the reconstruction error (difference between curves b and c). For each patients, curves b matched their corresponding theoretical curve a. The highest variations corresponded to early diastole. The maximum differences between the gold standard and the mean of the 100 reconstructions are presented for each patient in Table 3. The mean of these biases in absolute value was 6.1 cm/s with Feinstein model and 2.6 cm/s with the best two-phase model. The relation between time displacements and velocity biases is presented in Fig 5 (r^2^ = 0.7, p = 0.02).

**Table 3:** Maximum temporal displacements and velocity errors during Feinstein projection.

Patient	1	2	3	4	5	6	7
Time displacement (ms)	44.2	46.9	21.6	30.9	82.4	22.2	32.7
Velocity error (cm/s)	6.3	9.1	3.0	5.7	9.2	2.7	6.4

### High-resolution PC cine MRI data

**Fig 6:**
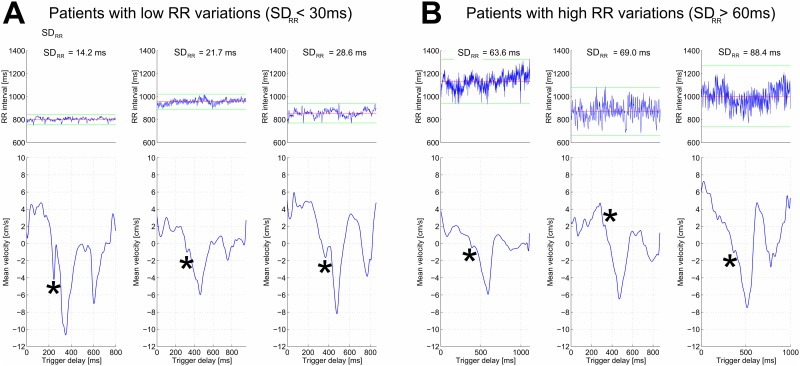
Tissue phase mapping velocity curves obtained from healthy volunteers with (A) low RR variations and (B) high RR variations. The graphs on the top line represent the cardiac cycle duration (Y-axis is the RR interval) during all the acquisition (X-axis is the position of the cardiac cycle during the acquisition). The velocity curves are on the bottom line and the star symbols (*) indicate the isovolumic relaxation peaks. These peaks are more precise (higher peaks) in the group with lower RR variability. SD_RR_ is the standard deviation of cardiac cycle durations during the acquisition.

Among the volunteers who underwent MRI, the heart rate variability differed with RR interval standard deviations (SD_RR_) ranging from 14.2 ms to 88.4 ms (Fig 6 top). The three volunteers with lowest (respectively highest) RR variability had SD_RR_ < 30 ms (respectively SD_RR_ > 60 ms). The myocardial velocity curves obtained from the volunteers with low or high RR variations are presented in Fig 6 (bottom). The heights of the short isovolumic relaxation peaks (R wave) were higher for the volunteers whose heart rates were more stable (p = 0.05).

## Discussion

The main interest of this study was to prove that heart rate variability can impair the temporal resolution of cine MRI acquisition. We have assessed the influence on temporal resolution of Feinstein’s model when it is used to combine k-space lines acquired during cardiac cycles of unequal lengths. The temporal misalignments inherent to this model have been described and quantified using a population of young children and adults without pathological arrhythmia. This population was chosen because higher beat-to-beat heart rate variations were expected (paediatric sinusal arrhythmia). Therefore our measurements constitute a kind of worst case. Patients with pathological arrhythmia were not chosen because in such situations the movement of the heart may be totally different from one beat to another (for example, the ventricle ejection fraction of a ventricular extrasystole is altered). In our study, the temporal misalignments reached 60 ms which is comparable to the temporal resolution of most clinical examinations (for example with TR = 4ms and VPS = 15, TR*VPS = 60ms). Therefore, these temporal misalignments should be taken into account when higher or equal temporal resolutions are achieved such as in many recent publications which often report temporal resolution lower than 30ms and sometimes close to 10ms [3–5,20–22]. The significance of such high temporal resolutions should be put into perspective because these temporal misalignments during the reconstruction process were generally not taken into account in those studies. The effective temporal resolution of a given sequence indeed depends on the acquisition speed (time needed to read a single k-space trajectory) but also on the reconstruction strategy (model used to combine data obtained during different cardiac cycles).

When the acquisition speed is very high, temporal misalignments result in many k-space lines associated with incorrect phases (or images). The consequence on the image is difficult to predict in general because it depends on which parts of the k-space have been corrupted. We chose to illustrate these consequences with a numerical phantom simulating PC cine MRI acquisition and with a qualitative analysis of actual PC cine MRI data from volunteers. In particular, our simulation results showed a blurring effect on the velocity curve that tended to erase small velocity peaks such as the isovolumic relaxation peaks. The velocity curves obtained from volunteers showed similar modifications of those isovolumic relaxation peaks, the amplitude of which were significantly lower in the “high heart rate variation” group. This finding is consistent with recent studies comparing tissue velocity measures from high-resolution PC cine MRI and from TDI, where MRI was found to slightly underestimate certain velocity peaks[4].

This study also aimed at analyzing which hypotheses underlying Feinstein’s model (the two-phase model and the estimation systole and diastole duration using Weissler’s formula) were responsible for most time misalignments. Interestingly, both assumptions were significantly involved in the misalignments. However, Weissler’s formula was relatively robust in our study and was responsible for only one third of the misalignments (10ms among 30ms) whereas the use of a two-phase model was responsible for two-third of the misalignments (20ms among 30ms). To allow higher temporal resolution to be achieved effectively, three solutions could be used to limit the temporal misalignments: 1/A better prediction of systole duration would only marginally reduce the time misalignments. The potential gain of such a solution is illustrated in the rightmost column of Fig 4. Although limited, such gain would probably be the easier to achieve. The impact of such gain on the velocity measurement has not been assessed in this study. 2/The method used to project a given cycle onto a mean cycle could be refined. A projection based on a 6- or 7-phase cardiac model instead of a 2-phase model could reduce misalignments; recently, two studies proposed to use real-time, low spatial resolution, PC MRI to identify the different cardiac phases and their variation as a function of the heart rate [17,23]. This could lead to the assessment of subject-specific cardiac models. 3/The use of external sensors (such as echocardiography) or internal navigators (low-spatial and high-temporal resolution acquisition) could allow a better estimation of the effective position within the cardiac cycle and subsequently could reduce the misalignments [24]. The residual time misalignments provided by such techniques still have to be determined.

### Limitations

1/This study was based on 306 cardiac cycles acquired in a population of children and young adults aged of 3–20 years. These patients were younger than most of the patients usually addressed to CMR. Therefore their baseline levels of sinusal arrhythmia can be expected to be higher than these observed during typical CMR examinations. 2/This study was based on classical segmented cine imaging with Cartesian k-space trajectories. Its conclusions cannot be straight forward transposed to new complex acquisition strategies which do not use Feinstein’s model, such as reconstruction which use low resolution images for cardiac phase recognition or k-t based reconstructions (which have specific temporal frequency underlying hypothesis)[25].

## Conclusion

We have assessed the influence of the temporal projection used during cine MRI reconstruction and proved that the conventionally used Feinstein model may induce time misalignments close to 30–60ms. Whenever high temporal resolution is desired, these time misalignments should be considered as they are likely to lower the effective temporal resolution, especially in patients with high heart rate variability such as children and young adults.

## Supporting Information

S1 Fig Tissue phase mapping MRI simulations with acquisition spanned over different cycles for every patients and using the two tested models (Feinstein model on the left) and the best two-phase model on the right. These figures comprise four lines: (A) velocity template used for the simulation, (B) acquisition without heart beat variations, (C) acquisition with heart beat variation, (D) error due to heart beat variation.
(ZIP)

S1 File TDI images acquired from the children and young adult population. (ZIP)
