# Supplementary figures and images for: Is High Temporal Resolution Achievable for Paediatric Cardiac Acquisitions during Several Heart Beats? Illustration with Cardiac Phase Contrast Cine-MRI (part 1 of 2)
Source: PLoS One. 2015 Nov 24;10(11):e0143744. doi: 10.1371/journal.pone.0143744 (PMC4658039; doi:10.1371/journal.pone.0143744)

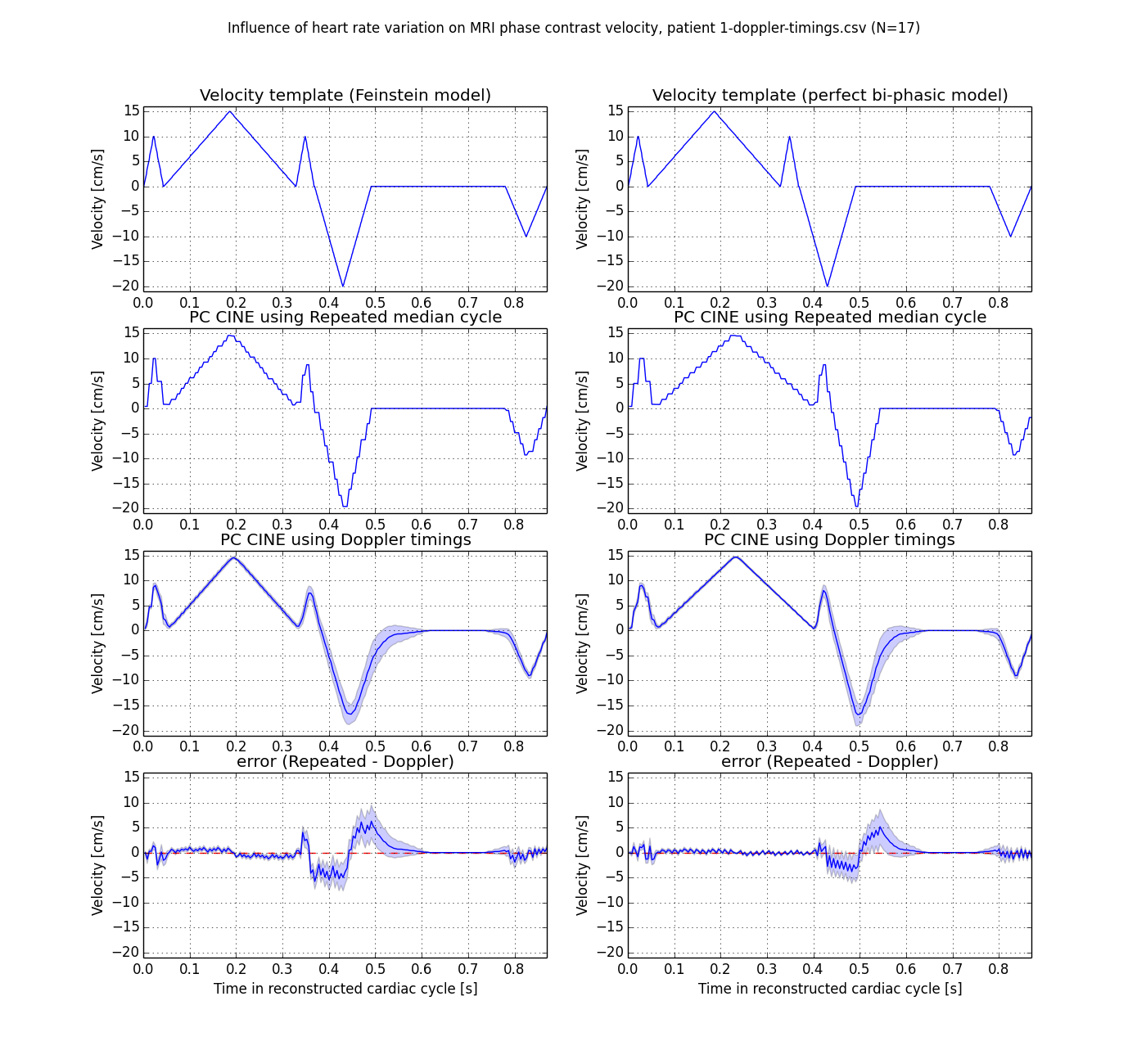

Supplement: S1 Fig — These figures comprise four lines: (A) velocity template used for the simulation, (B) acquisition without heart beat variations, (C) acquisition with heart beat variation, (D) error due to heart beat variation. (ZIP) [file pone.0143744.s001.zip › modsim_1.tif]

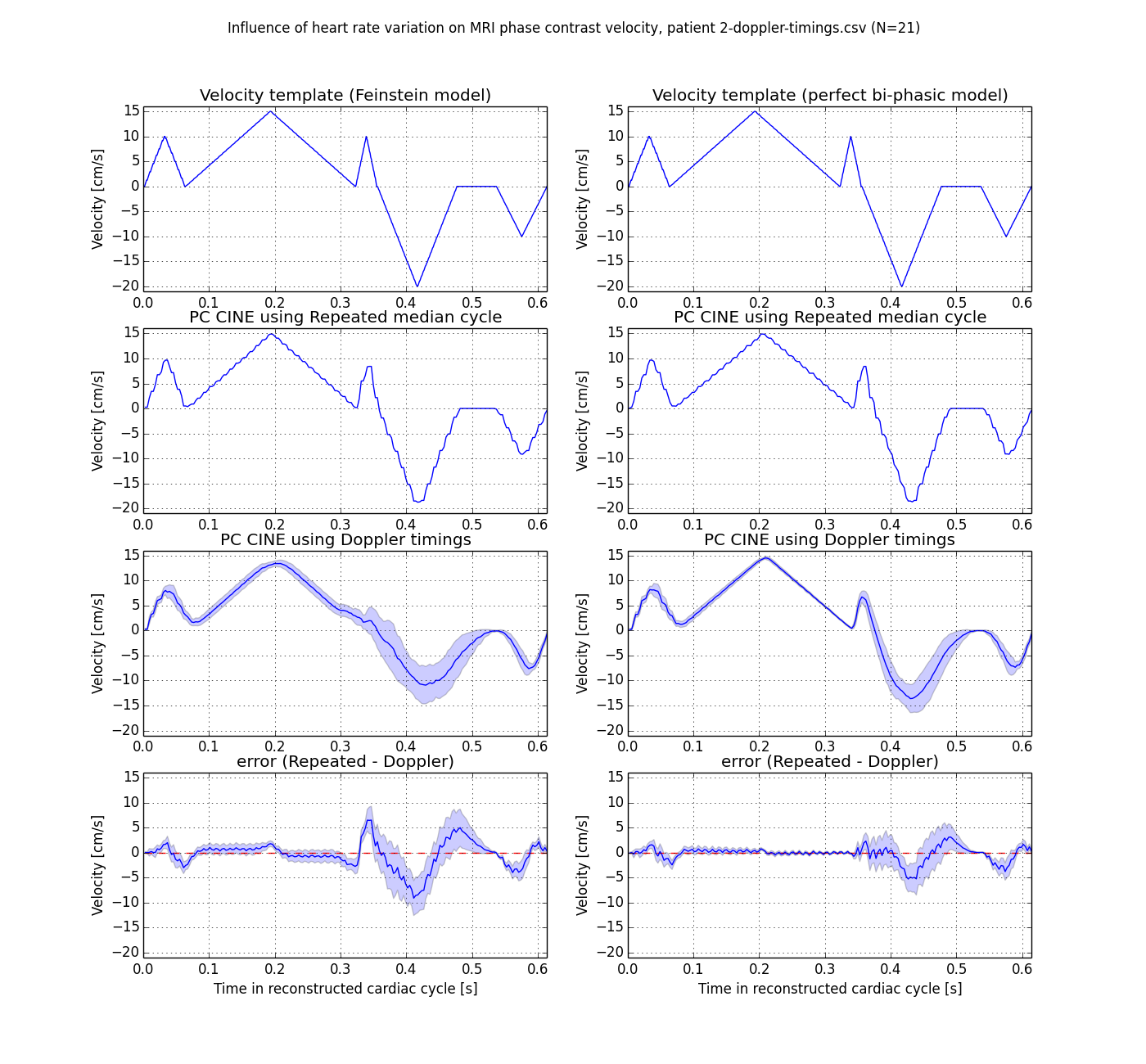

Supplement: S1 Fig — These figures comprise four lines: (A) velocity template used for the simulation, (B) acquisition without heart beat variations, (C) acquisition with heart beat variation, (D) error due to heart beat variation. (ZIP) [file pone.0143744.s001.zip › modsim_2.tif]

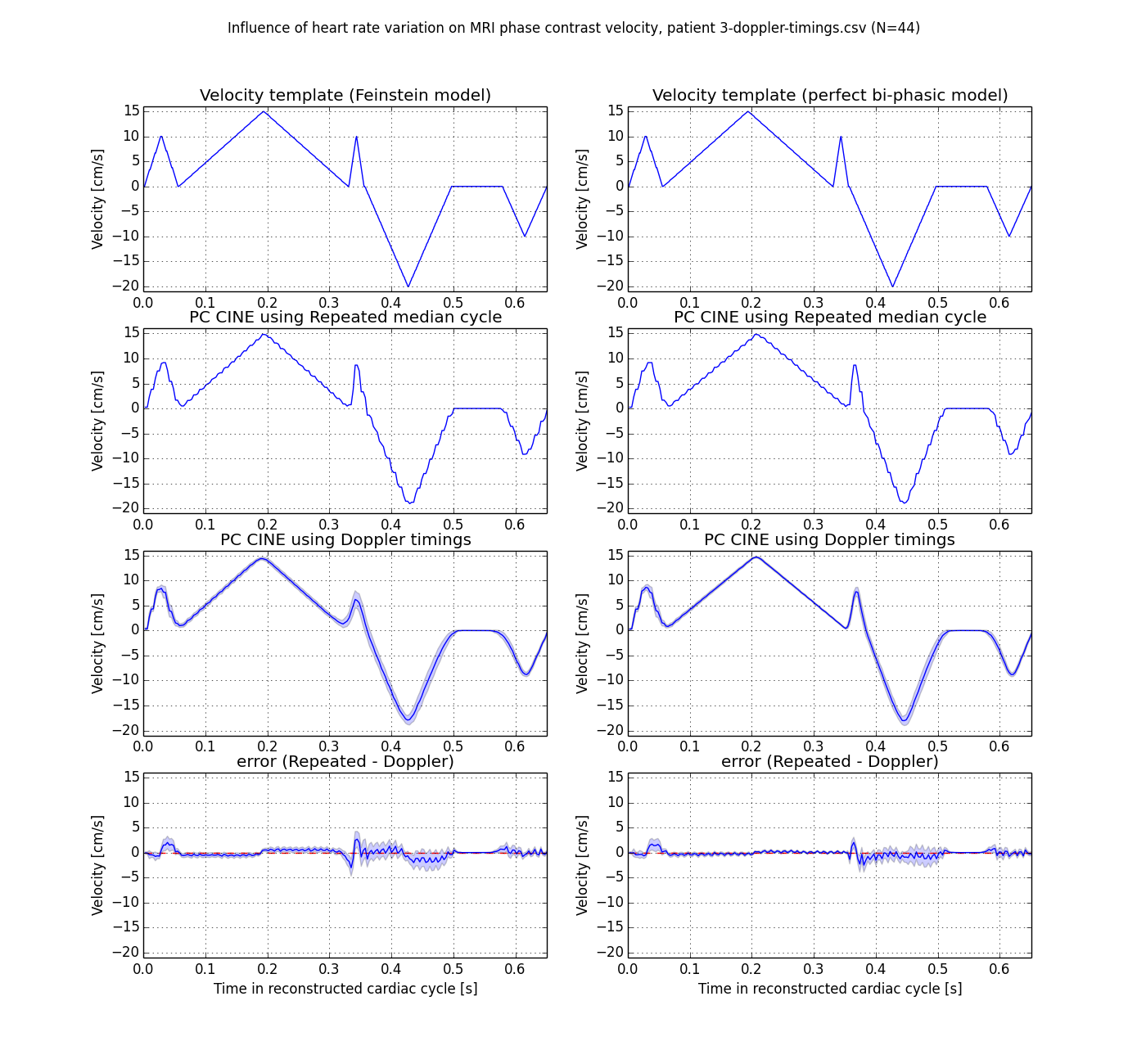

Supplement: S1 Fig — These figures comprise four lines: (A) velocity template used for the simulation, (B) acquisition without heart beat variations, (C) acquisition with heart beat variation, (D) error due to heart beat variation. (ZIP) [file pone.0143744.s001.zip › modsim_3.tif]

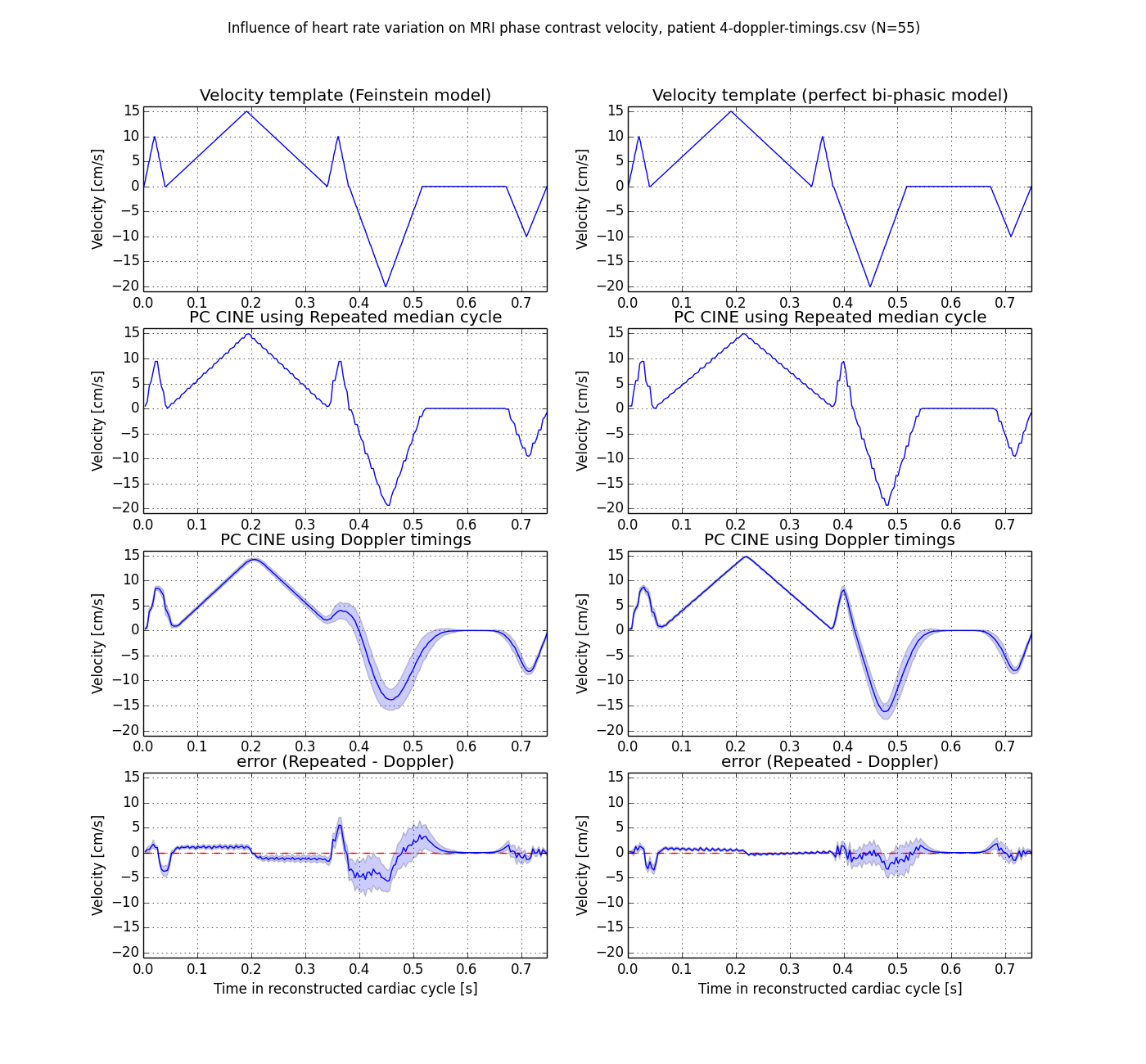

Supplement: S1 Fig — These figures comprise four lines: (A) velocity template used for the simulation, (B) acquisition without heart beat variations, (C) acquisition with heart beat variation, (D) error due to heart beat variation. (ZIP) [file pone.0143744.s001.zip › modsim_4.tif]

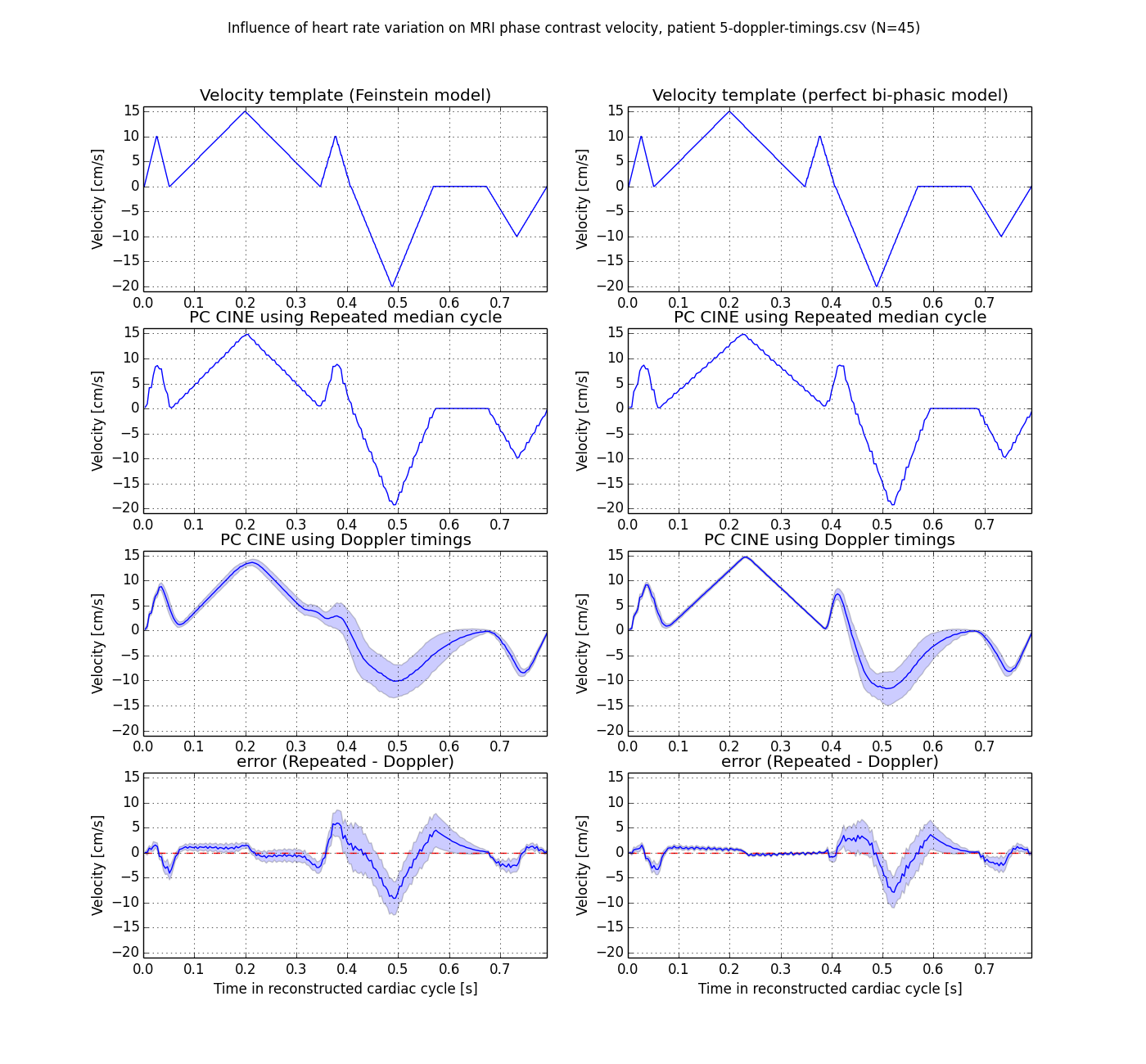

Supplement: S1 Fig — These figures comprise four lines: (A) velocity template used for the simulation, (B) acquisition without heart beat variations, (C) acquisition with heart beat variation, (D) error due to heart beat variation. (ZIP) [file pone.0143744.s001.zip › modsim_5.tif]

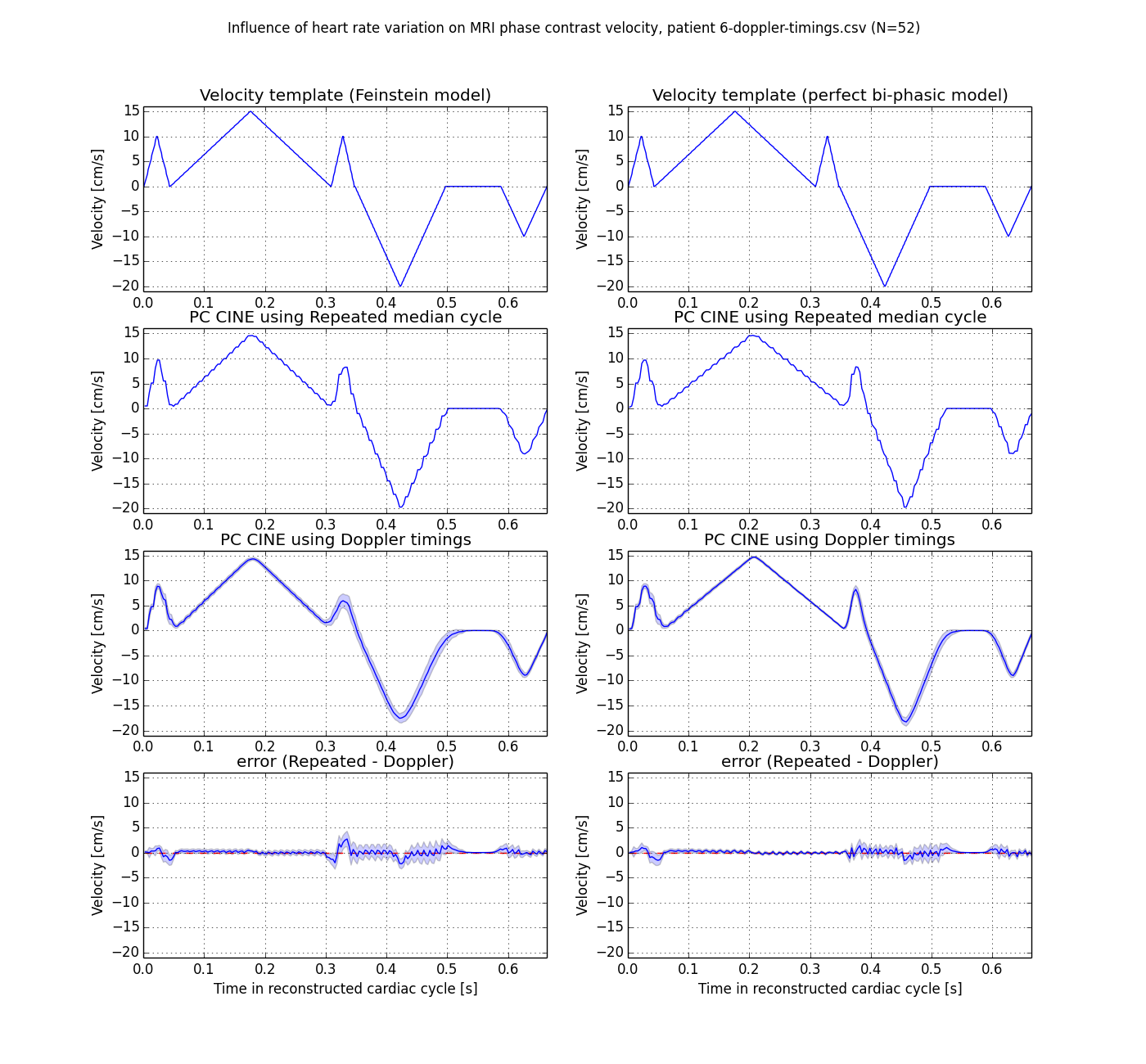

Supplement: S1 Fig — These figures comprise four lines: (A) velocity template used for the simulation, (B) acquisition without heart beat variations, (C) acquisition with heart beat variation, (D) error due to heart beat variation. (ZIP) [file pone.0143744.s001.zip › modsim_6.tif]

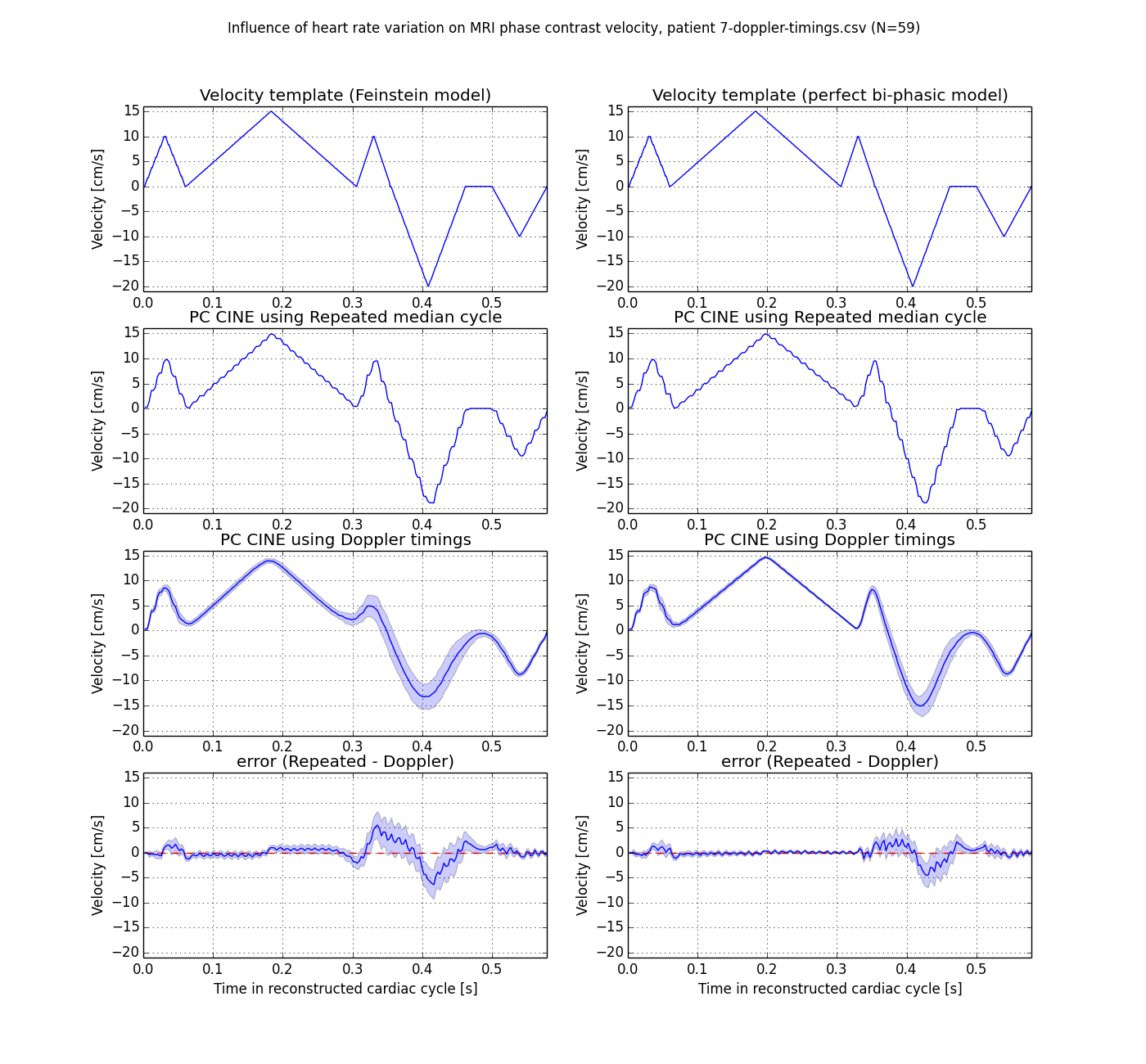

Supplement: S1 Fig — These figures comprise four lines: (A) velocity template used for the simulation, (B) acquisition without heart beat variations, (C) acquisition with heart beat variation, (D) error due to heart beat variation. (ZIP) [file pone.0143744.s001.zip › modsim_7.tif]

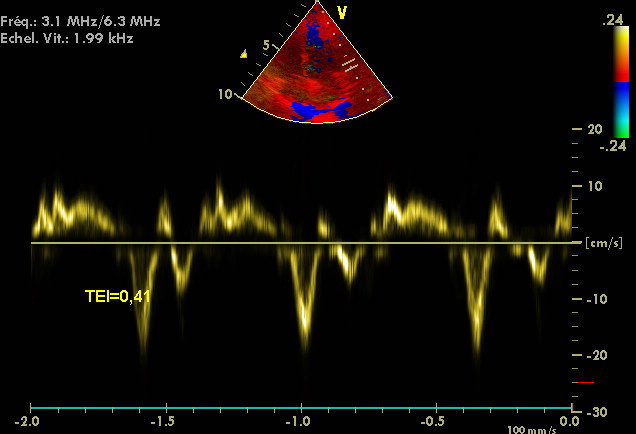

Supplement: S1 File — (ZIP) [file pone.0143744.s002.zip › Image01.jpg]

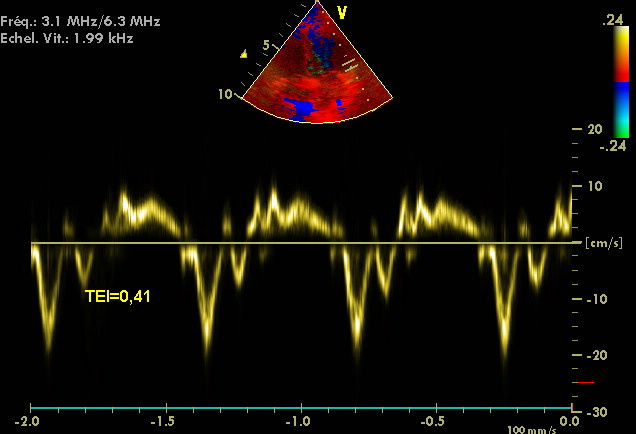

Supplement: S1 File — (ZIP) [file pone.0143744.s002.zip › Image04.jpg]

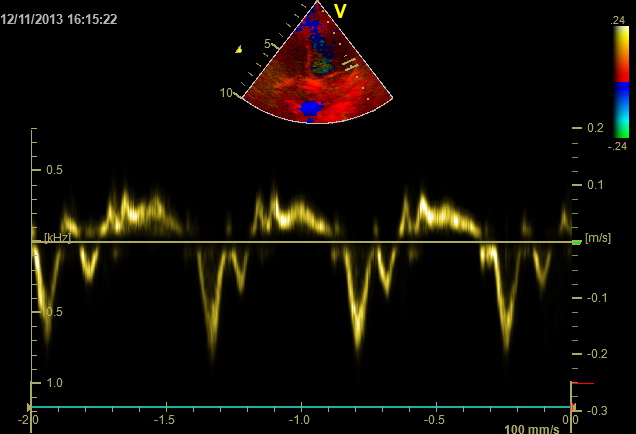

Supplement: S1 File — (ZIP) [file pone.0143744.s002.zip › Image05.jpg]

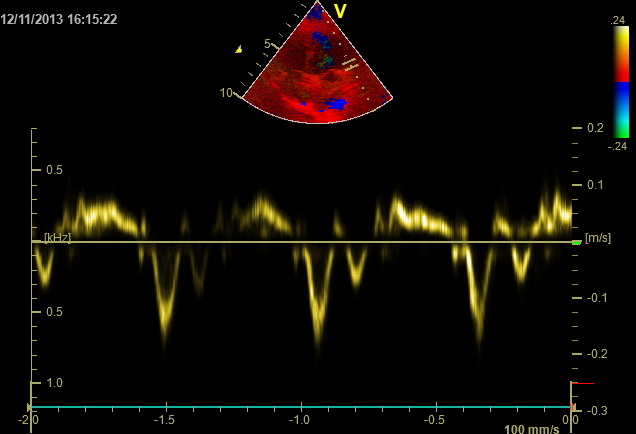

Supplement: S1 File — (ZIP) [file pone.0143744.s002.zip › Image06.jpg]

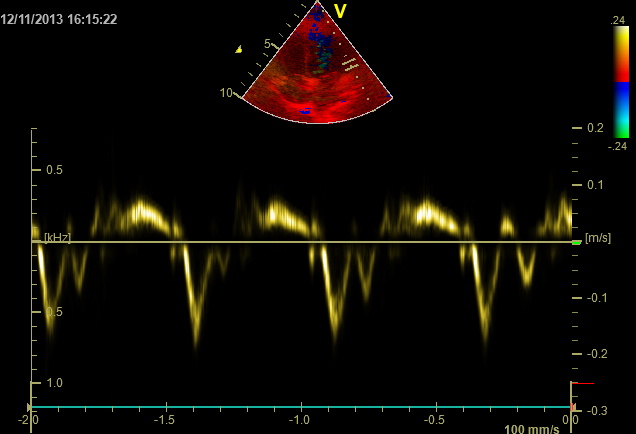

Supplement: S1 File — (ZIP) [file pone.0143744.s002.zip › Image07.jpg]

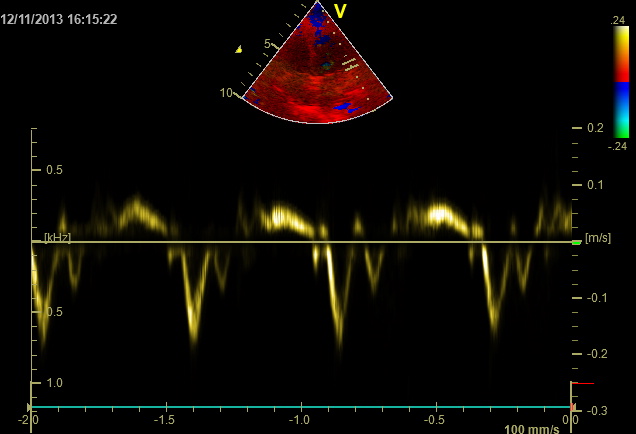

Supplement: S1 File — (ZIP) [file pone.0143744.s002.zip › Image08.jpg]

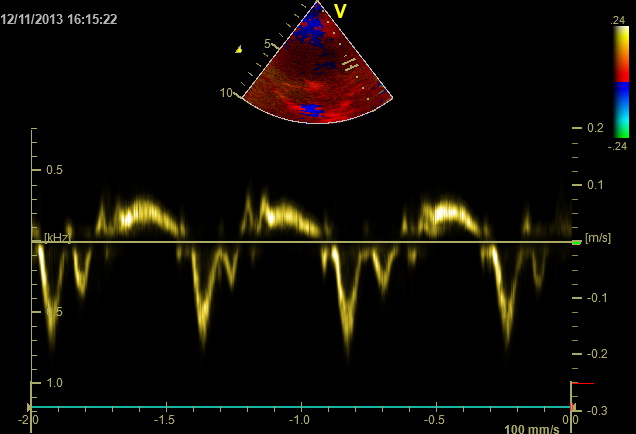

Supplement: S1 File — (ZIP) [file pone.0143744.s002.zip › Image09.jpg]

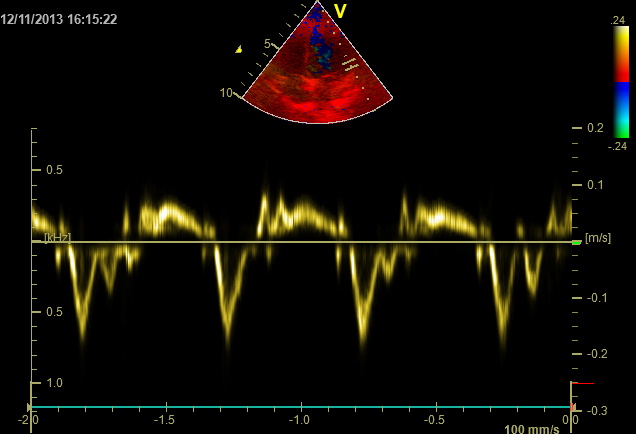

Supplement: S1 File — (ZIP) [file pone.0143744.s002.zip › Image10.jpg]

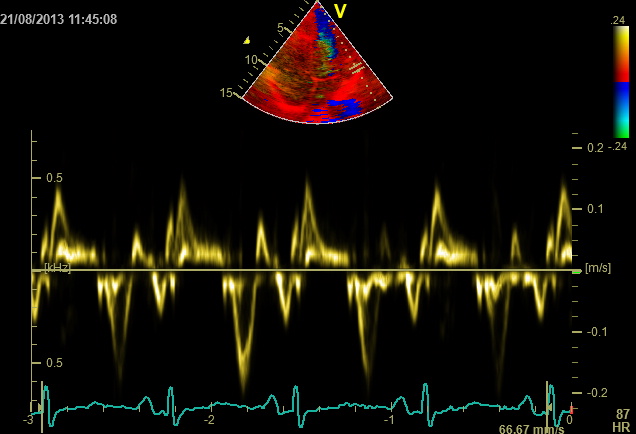

Supplement: S1 File — (ZIP) [file pone.0143744.s002.zip › Image101.jpg]

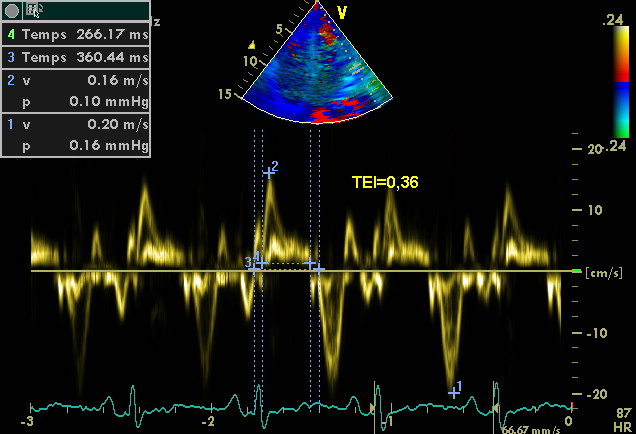

Supplement: S1 File — (ZIP) [file pone.0143744.s002.zip › Image102.jpg]

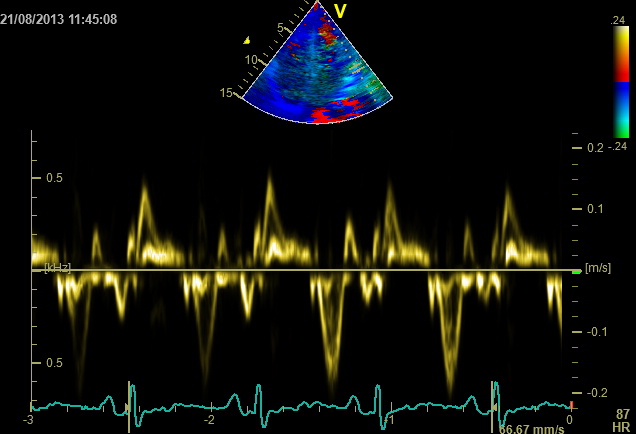

Supplement: S1 File — (ZIP) [file pone.0143744.s002.zip › Image103.jpg]

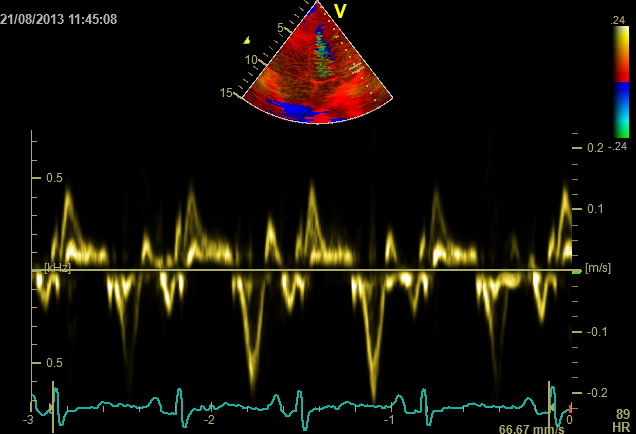

Supplement: S1 File — (ZIP) [file pone.0143744.s002.zip › Image104.jpg]

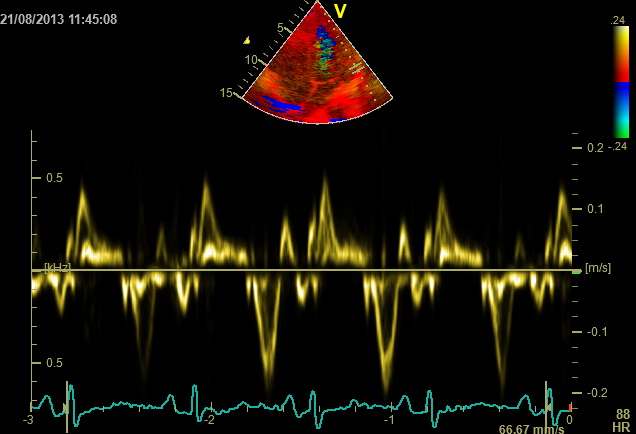

Supplement: S1 File — (ZIP) [file pone.0143744.s002.zip › Image105.jpg]

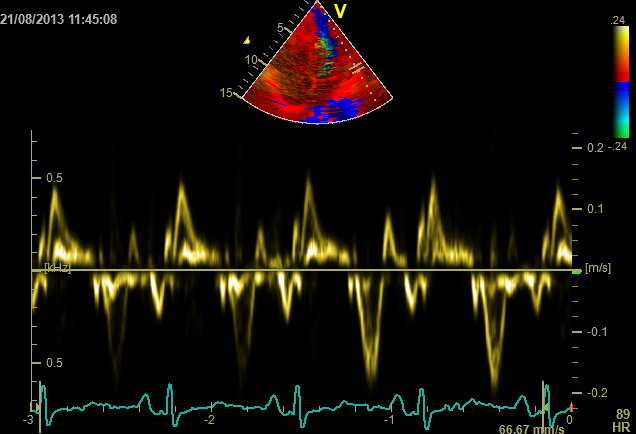

Supplement: S1 File — (ZIP) [file pone.0143744.s002.zip › Image106.jpg]

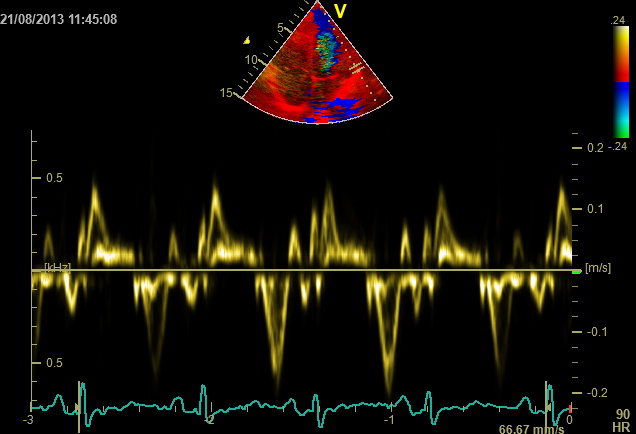

Supplement: S1 File — (ZIP) [file pone.0143744.s002.zip › Image107.jpg]

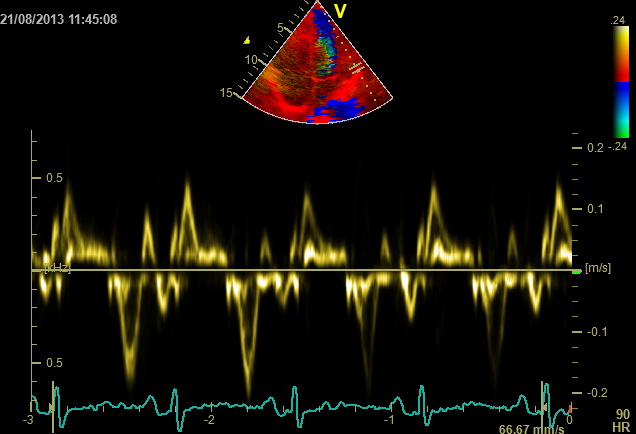

Supplement: S1 File — (ZIP) [file pone.0143744.s002.zip › Image108.jpg]

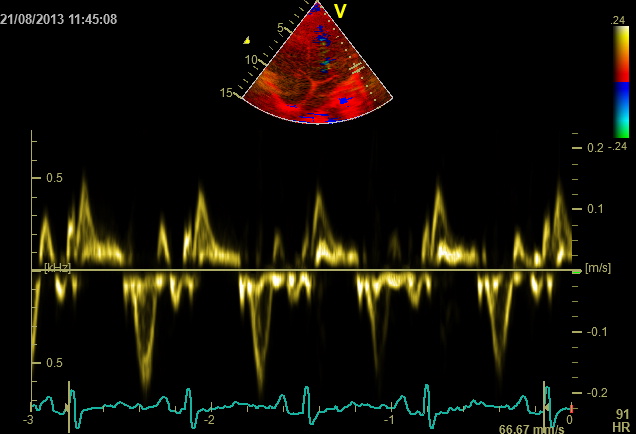

Supplement: S1 File — (ZIP) [file pone.0143744.s002.zip › Image109.jpg]

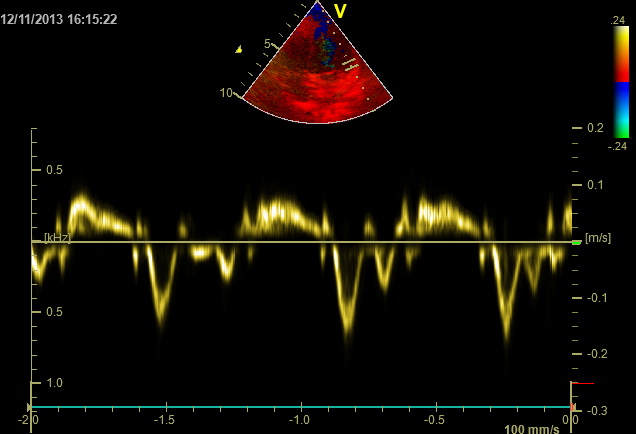

Supplement: S1 File — (ZIP) [file pone.0143744.s002.zip › Image11.jpg]

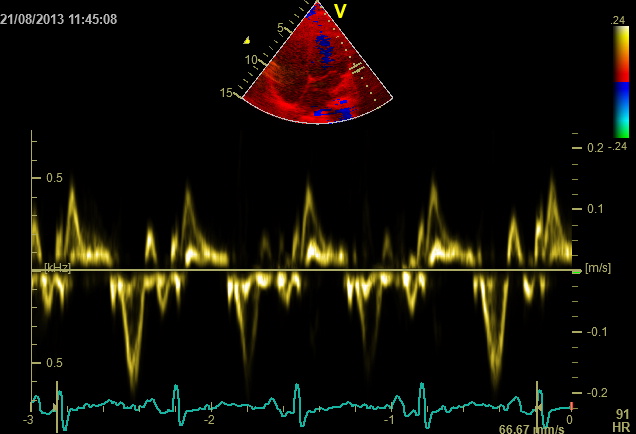

Supplement: S1 File — (ZIP) [file pone.0143744.s002.zip › Image111.jpg]

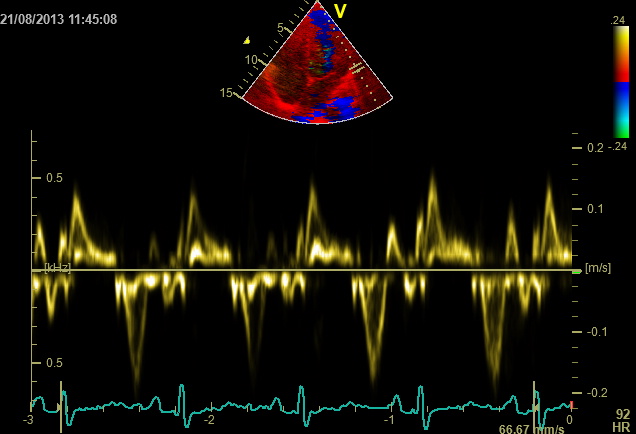

Supplement: S1 File — (ZIP) [file pone.0143744.s002.zip › Image112.jpg]

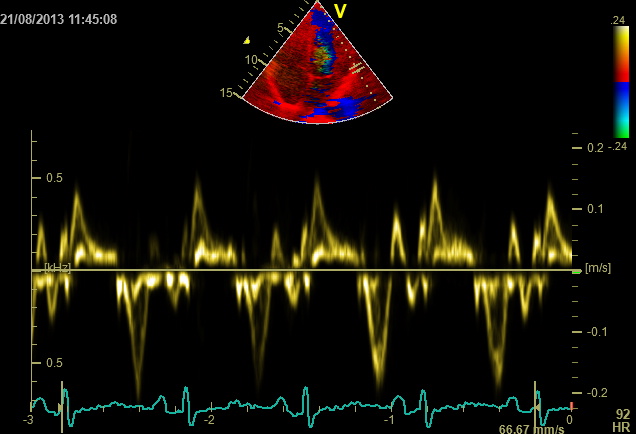

Supplement: S1 File — (ZIP) [file pone.0143744.s002.zip › Image113.jpg]

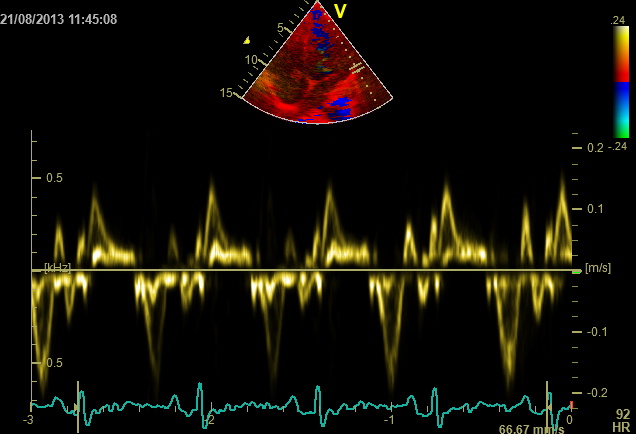

Supplement: S1 File — (ZIP) [file pone.0143744.s002.zip › Image114.jpg]

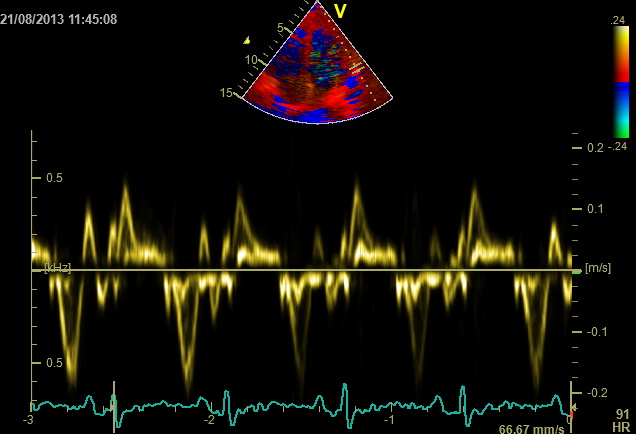

Supplement: S1 File — (ZIP) [file pone.0143744.s002.zip › Image115.jpg]

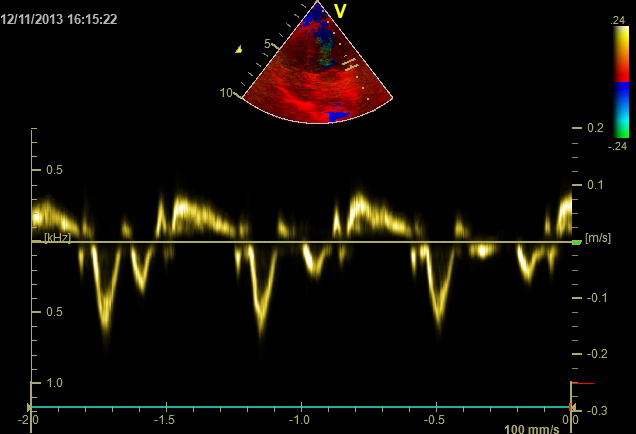

Supplement: S1 File — (ZIP) [file pone.0143744.s002.zip › Image12.jpg]

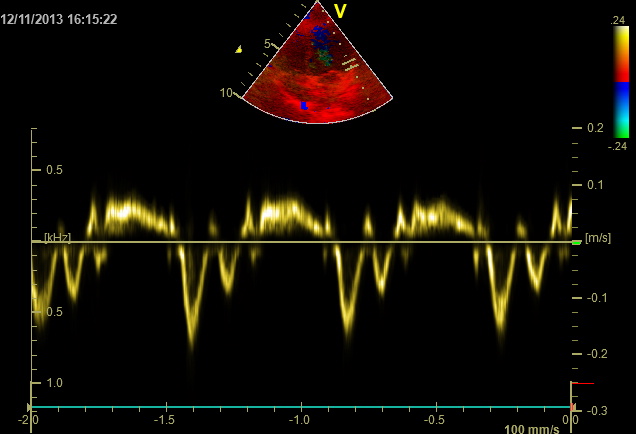

Supplement: S1 File — (ZIP) [file pone.0143744.s002.zip › Image13.jpg]

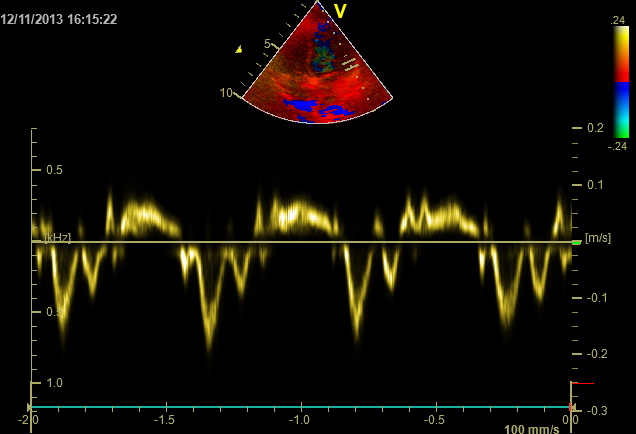

Supplement: S1 File — (ZIP) [file pone.0143744.s002.zip › Image14.jpg]

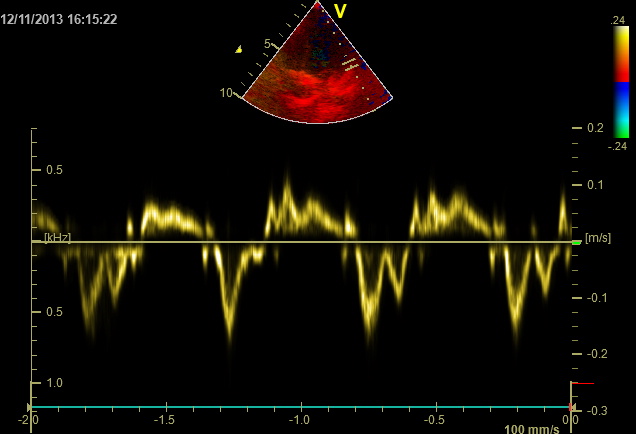

Supplement: S1 File — (ZIP) [file pone.0143744.s002.zip › Image15.jpg]

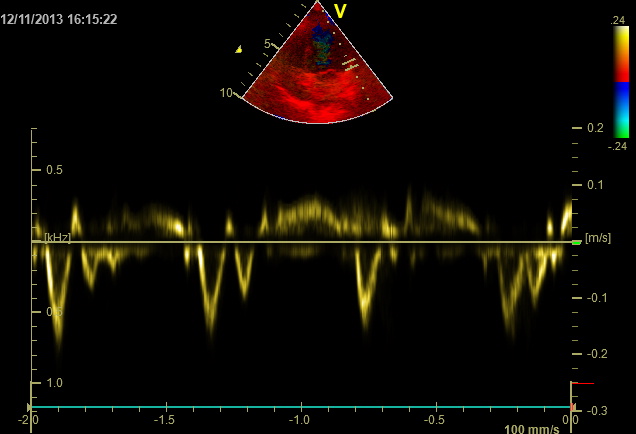

Supplement: S1 File — (ZIP) [file pone.0143744.s002.zip › Image16.jpg]

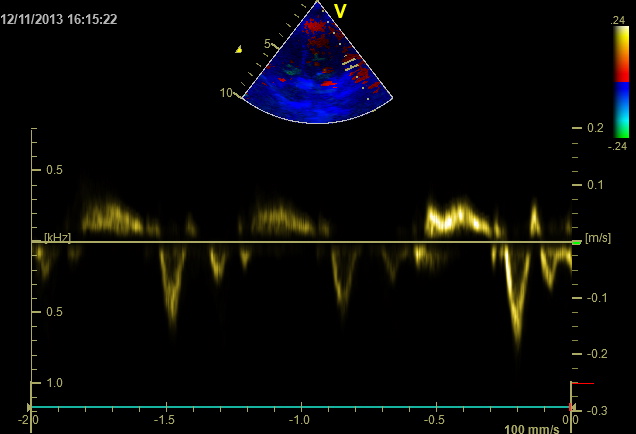

Supplement: S1 File — (ZIP) [file pone.0143744.s002.zip › Image17.jpg]

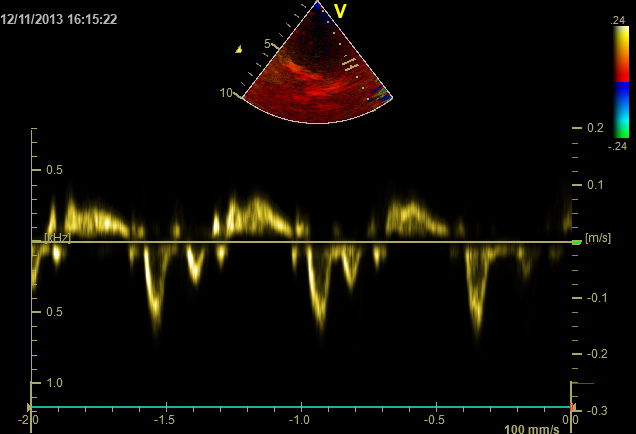

Supplement: S1 File — (ZIP) [file pone.0143744.s002.zip › Image18.jpg]

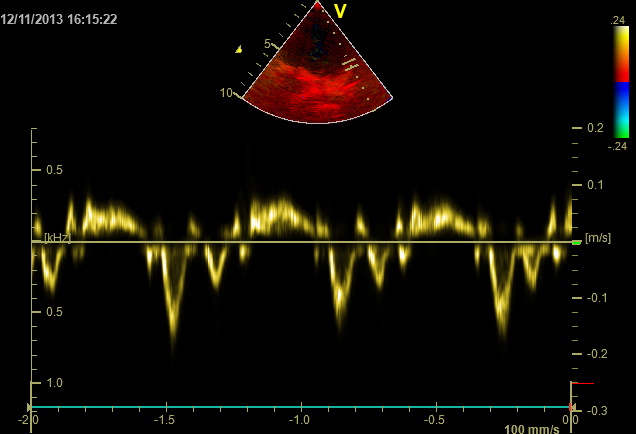

Supplement: S1 File — (ZIP) [file pone.0143744.s002.zip › Image19.jpg]

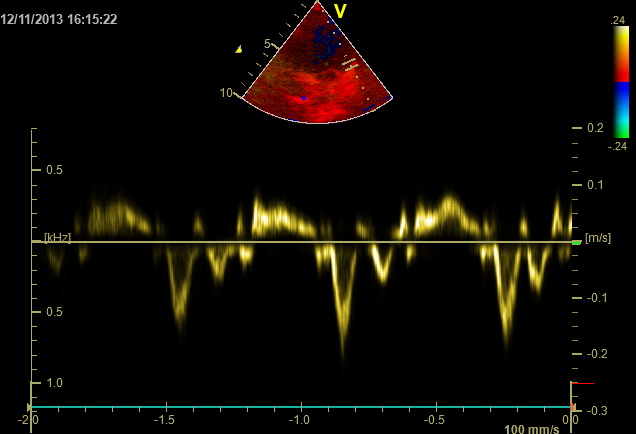

Supplement: S1 File — (ZIP) [file pone.0143744.s002.zip › Image20.jpg]

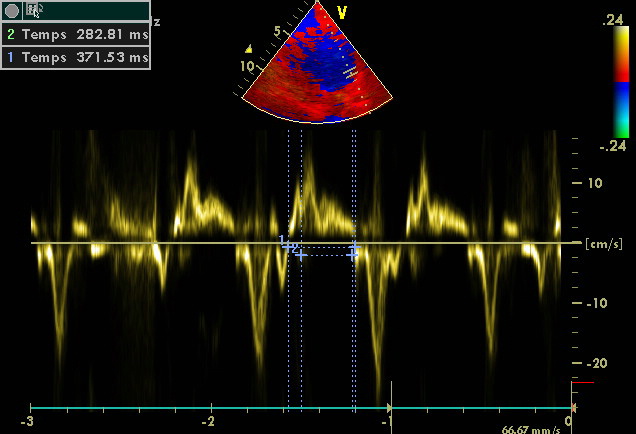

Supplement: S1 File — (ZIP) [file pone.0143744.s002.zip › Image201.jpg]

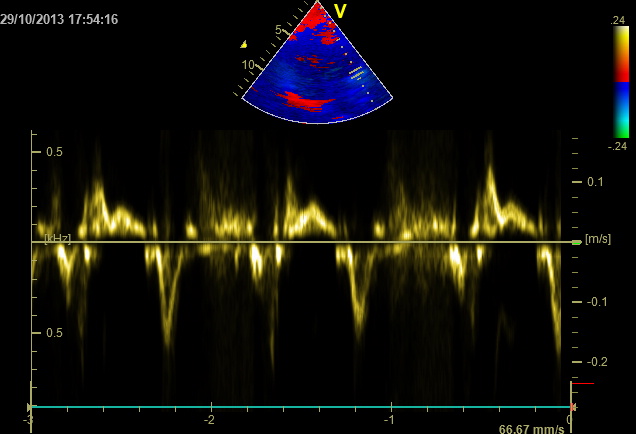

Supplement: S1 File — (ZIP) [file pone.0143744.s002.zip › Image203.jpg]

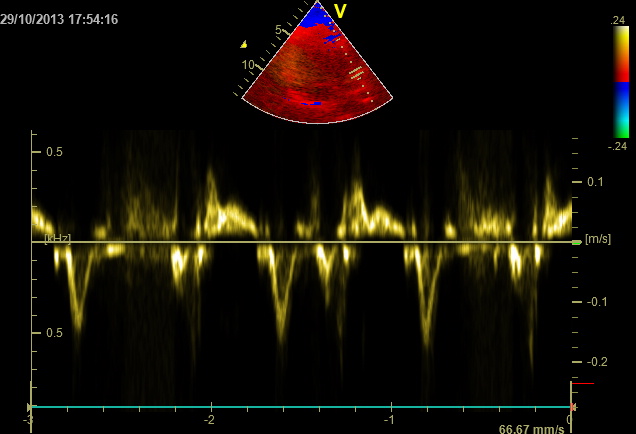

Supplement: S1 File — (ZIP) [file pone.0143744.s002.zip › Image204.jpg]

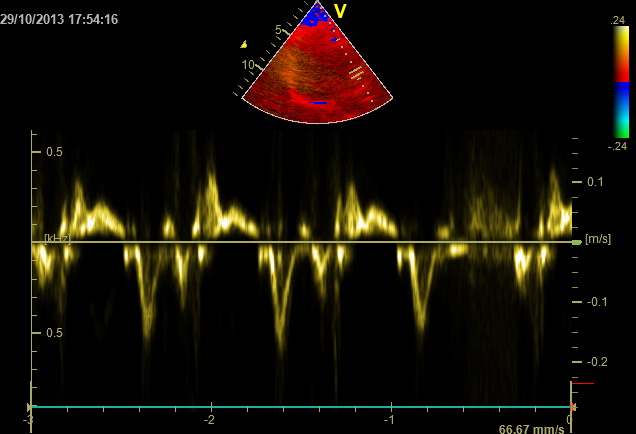

Supplement: S1 File — (ZIP) [file pone.0143744.s002.zip › Image205.jpg]

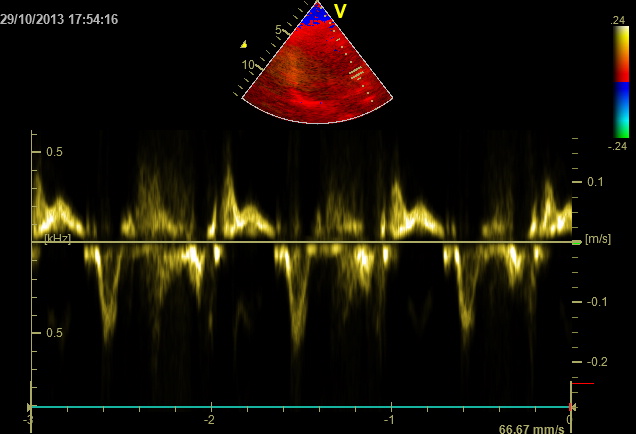

Supplement: S1 File — (ZIP) [file pone.0143744.s002.zip › Image206.jpg]

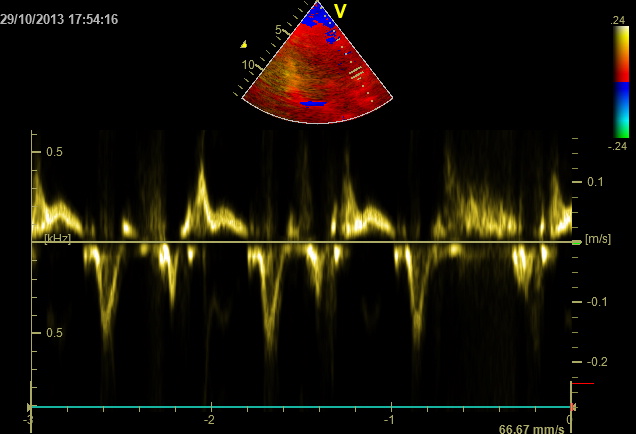

Supplement: S1 File — (ZIP) [file pone.0143744.s002.zip › Image207.jpg]

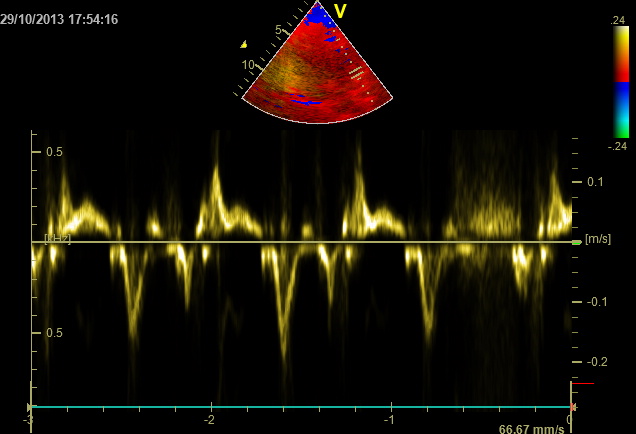

Supplement: S1 File — (ZIP) [file pone.0143744.s002.zip › Image208.jpg]

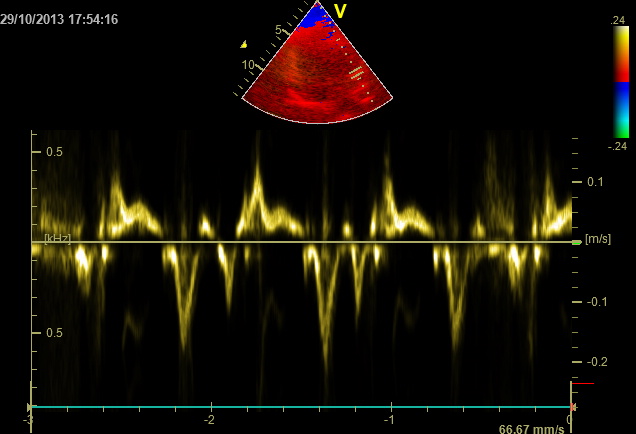

Supplement: S1 File — (ZIP) [file pone.0143744.s002.zip › Image209.jpg]

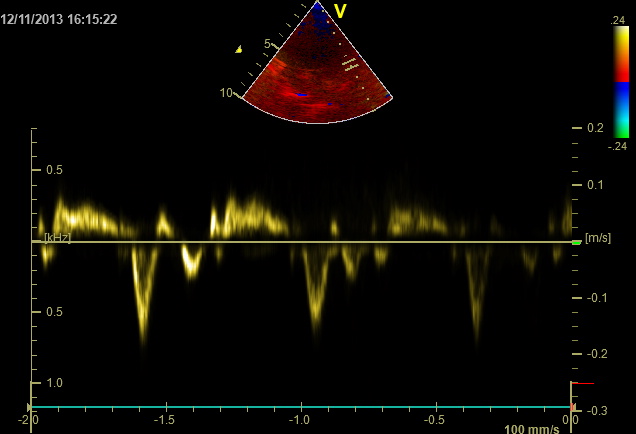

Supplement: S1 File — (ZIP) [file pone.0143744.s002.zip › Image21.jpg]

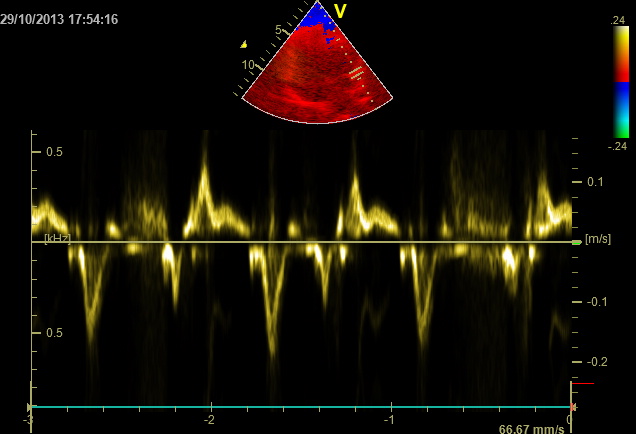

Supplement: S1 File — (ZIP) [file pone.0143744.s002.zip › Image210.jpg]

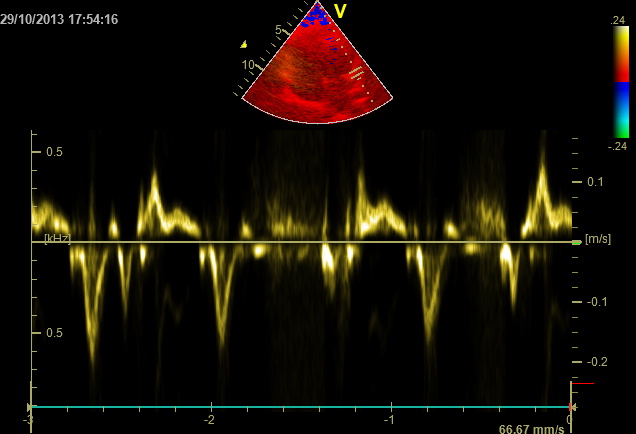

Supplement: S1 File — (ZIP) [file pone.0143744.s002.zip › Image211.jpg]

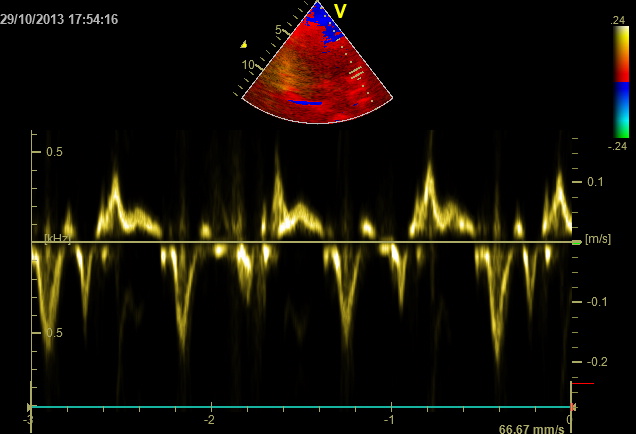

Supplement: S1 File — (ZIP) [file pone.0143744.s002.zip › Image212.jpg]

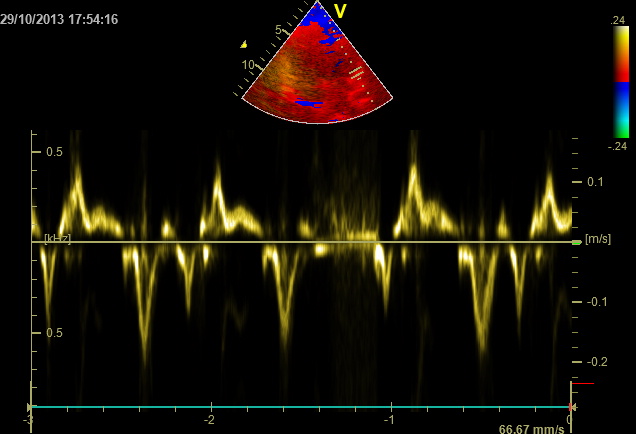

Supplement: S1 File — (ZIP) [file pone.0143744.s002.zip › Image213.jpg]

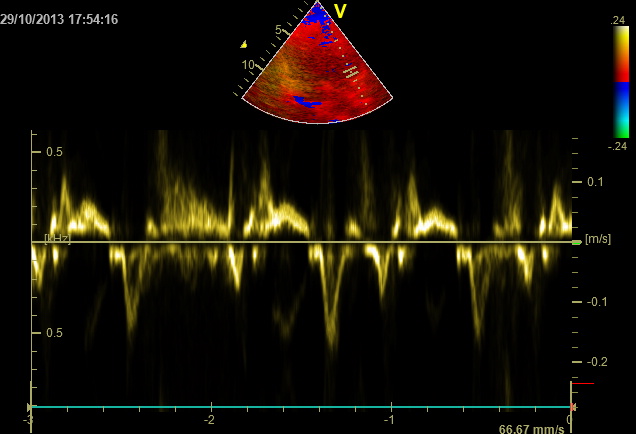

Supplement: S1 File — (ZIP) [file pone.0143744.s002.zip › Image214.jpg]

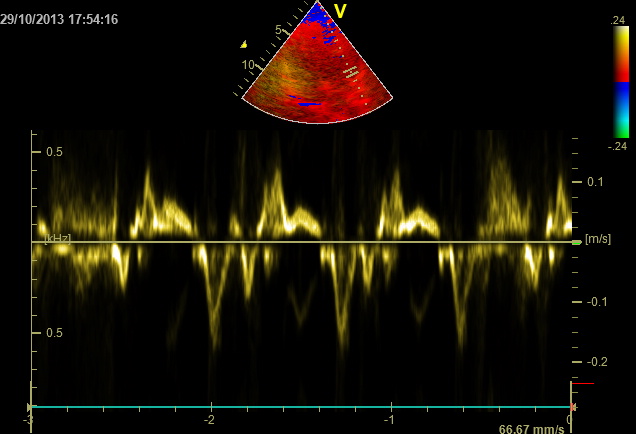

Supplement: S1 File — (ZIP) [file pone.0143744.s002.zip › Image215.jpg]

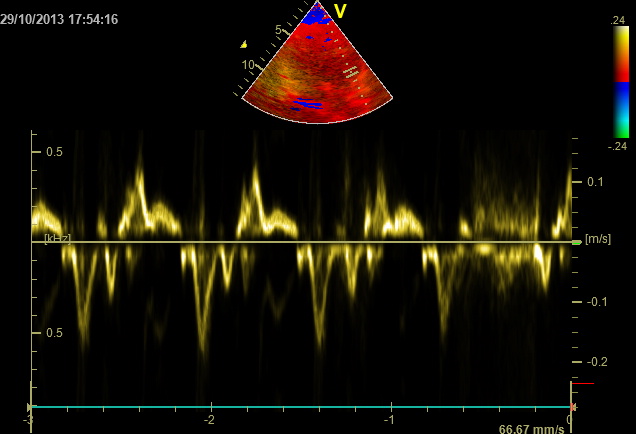

Supplement: S1 File — (ZIP) [file pone.0143744.s002.zip › Image216.jpg]

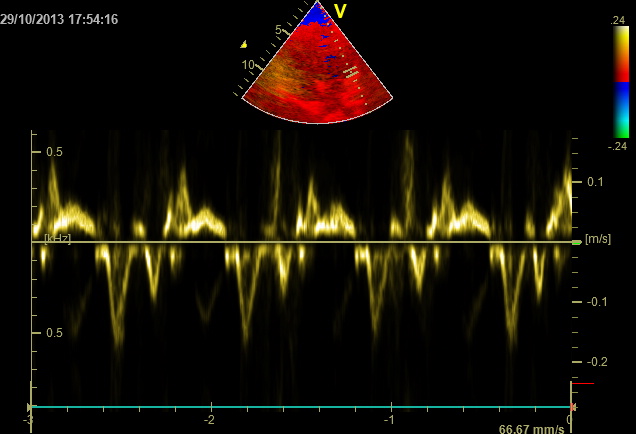

Supplement: S1 File — (ZIP) [file pone.0143744.s002.zip › Image217.jpg]

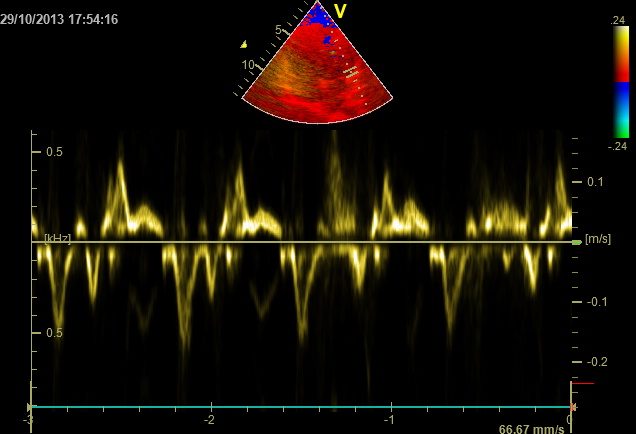

Supplement: S1 File — (ZIP) [file pone.0143744.s002.zip › Image218.jpg]

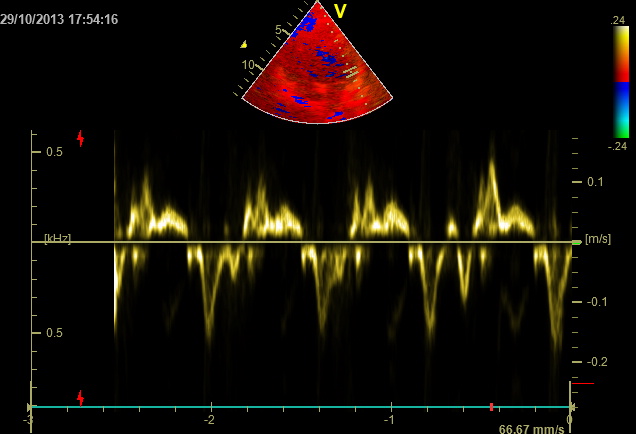

Supplement: S1 File — (ZIP) [file pone.0143744.s002.zip › Image219.jpg]

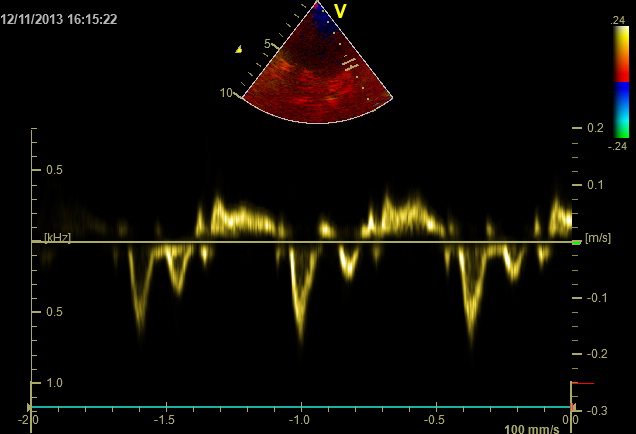

Supplement: S1 File — (ZIP) [file pone.0143744.s002.zip › Image22.jpg]

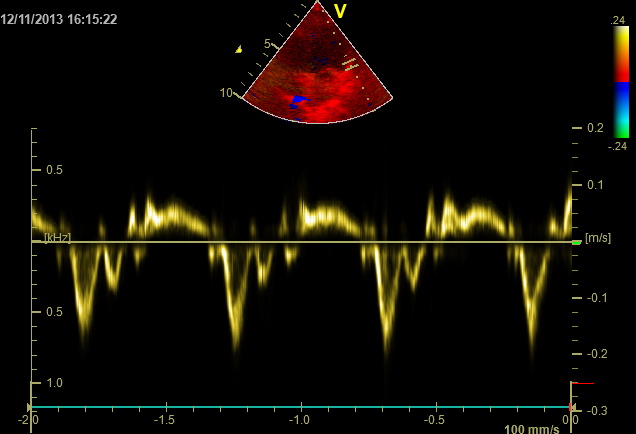

Supplement: S1 File — (ZIP) [file pone.0143744.s002.zip › Image24.jpg]

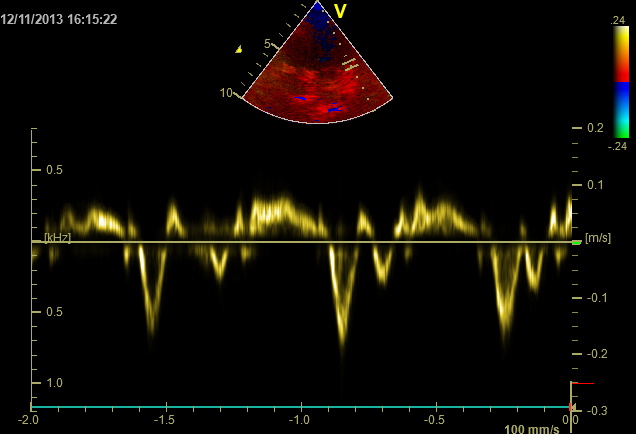

Supplement: S1 File — (ZIP) [file pone.0143744.s002.zip › Image25.jpg]

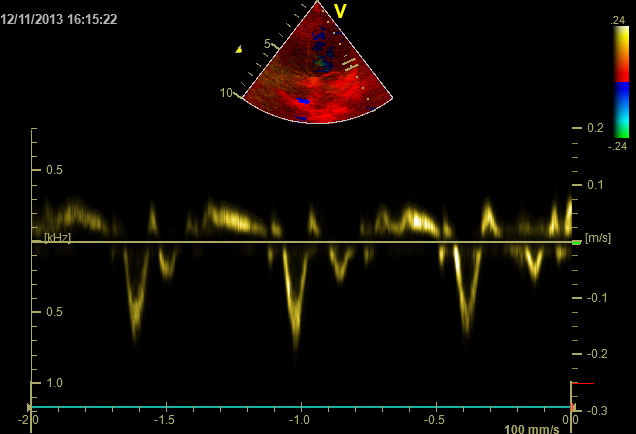

Supplement: S1 File — (ZIP) [file pone.0143744.s002.zip › Image26.jpg]

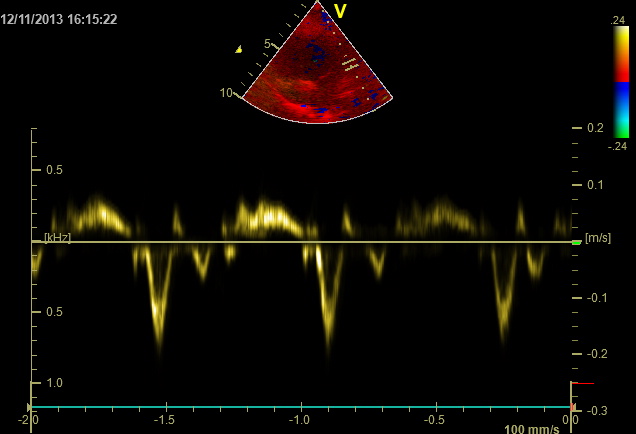

Supplement: S1 File — (ZIP) [file pone.0143744.s002.zip › Image28.jpg]

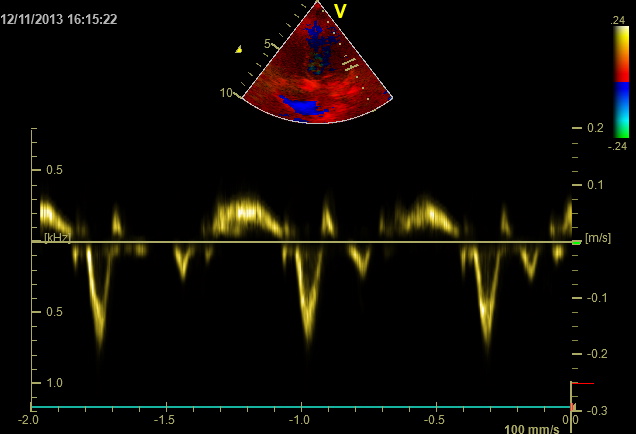

Supplement: S1 File — (ZIP) [file pone.0143744.s002.zip › Image29.jpg]

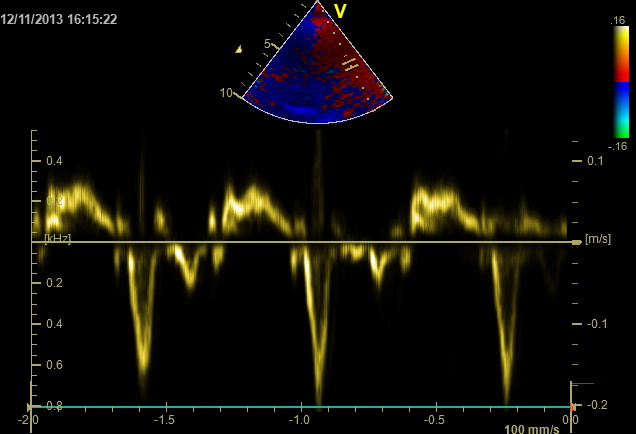

Supplement: S1 File — (ZIP) [file pone.0143744.s002.zip › Image30.jpg]

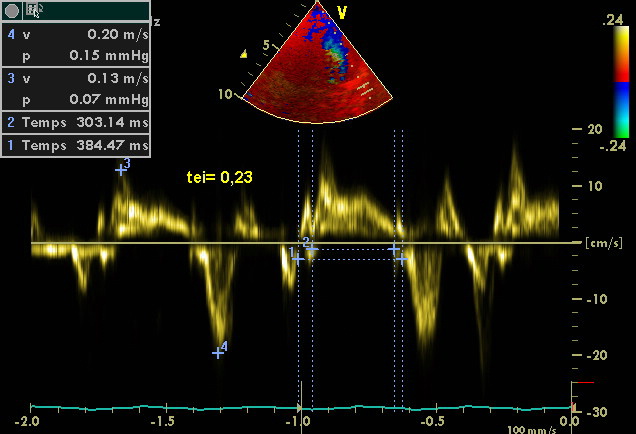

Supplement: S1 File — (ZIP) [file pone.0143744.s002.zip › Image301.jpg]

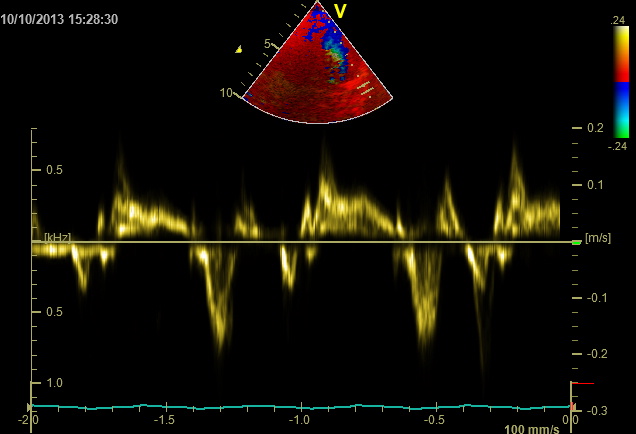

Supplement: S1 File — (ZIP) [file pone.0143744.s002.zip › Image302.jpg]

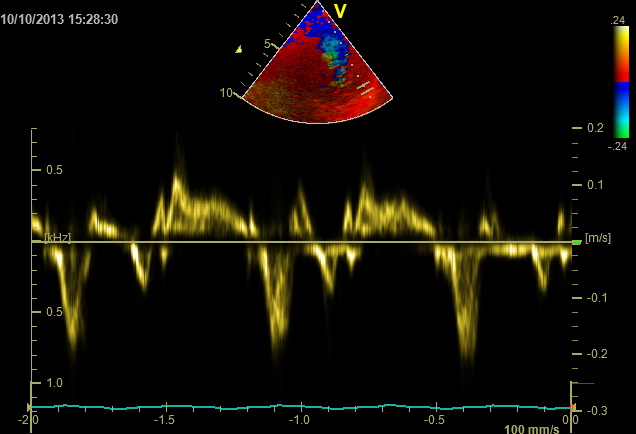

Supplement: S1 File — (ZIP) [file pone.0143744.s002.zip › Image303.jpg]

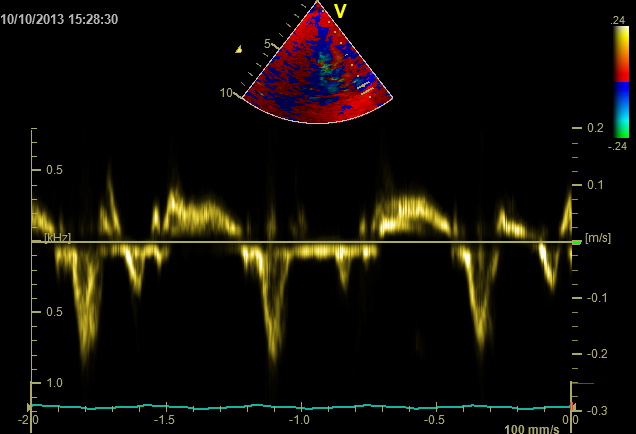

Supplement: S1 File — (ZIP) [file pone.0143744.s002.zip › Image304.jpg]

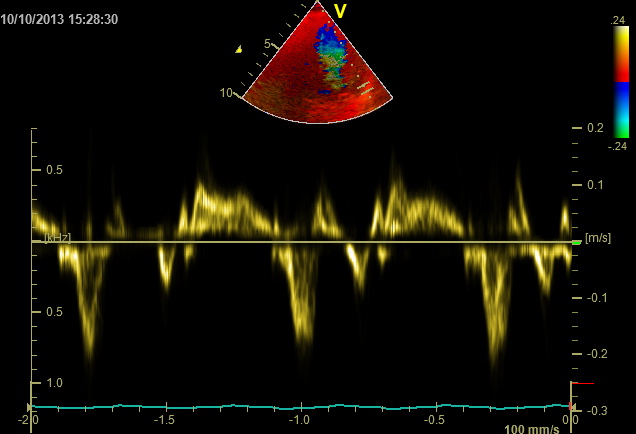

Supplement: S1 File — (ZIP) [file pone.0143744.s002.zip › Image305.jpg]

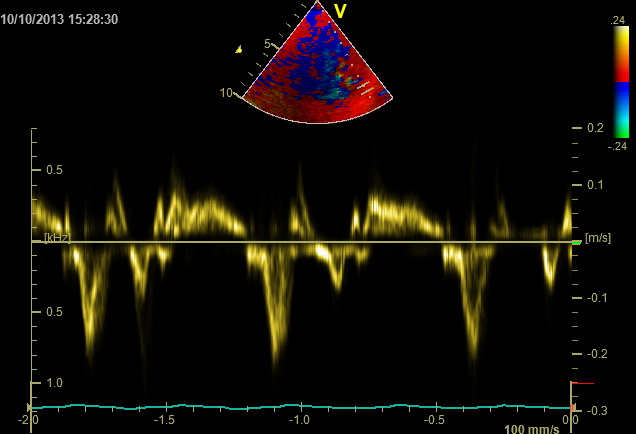

Supplement: S1 File — (ZIP) [file pone.0143744.s002.zip › Image306.jpg]

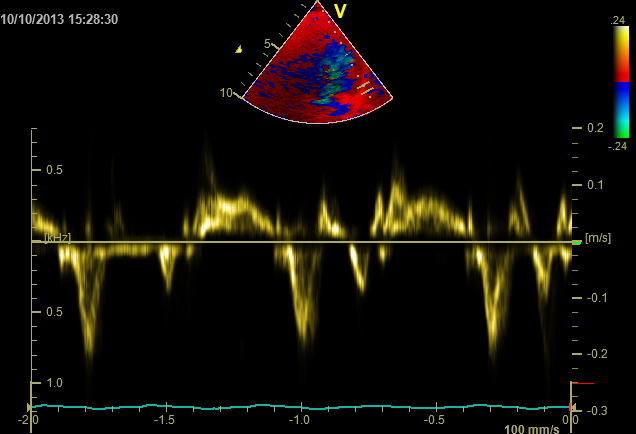

Supplement: S1 File — (ZIP) [file pone.0143744.s002.zip › Image307.jpg]

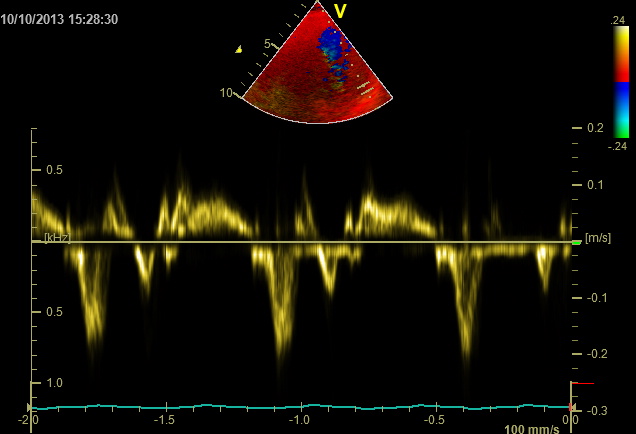

Supplement: S1 File — (ZIP) [file pone.0143744.s002.zip › Image308.jpg]

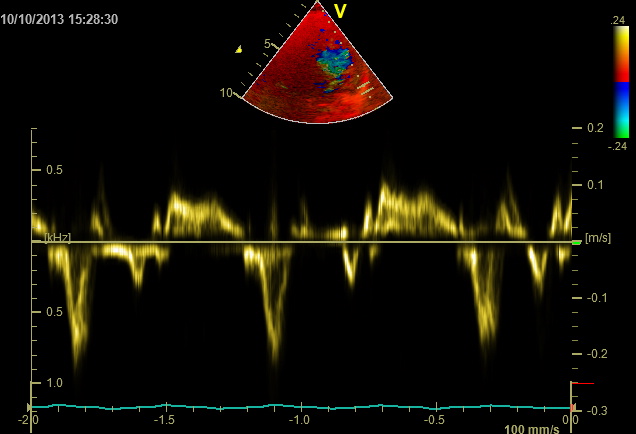

Supplement: S1 File — (ZIP) [file pone.0143744.s002.zip › Image309.jpg]

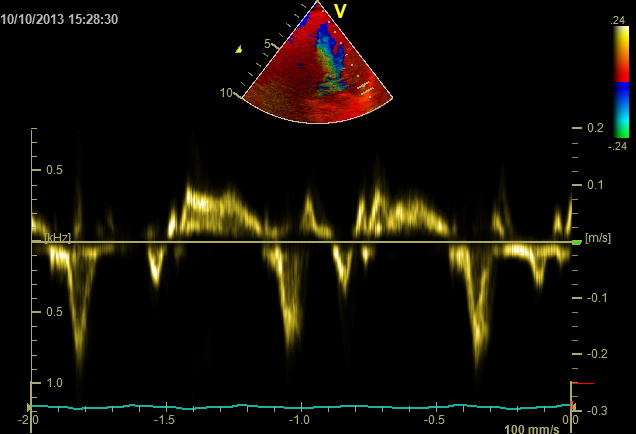

Supplement: S1 File — (ZIP) [file pone.0143744.s002.zip › Image310.jpg]

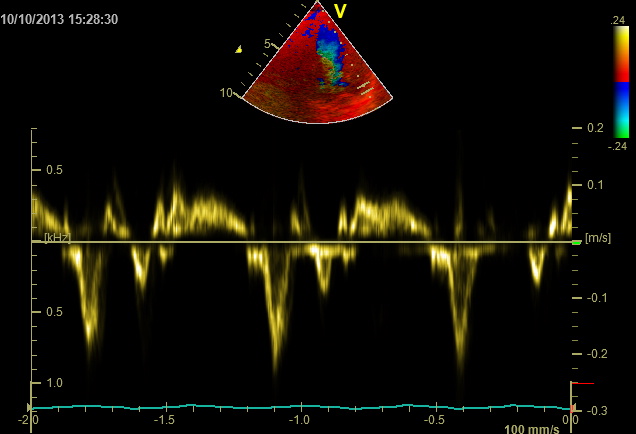

Supplement: S1 File — (ZIP) [file pone.0143744.s002.zip › Image311.jpg]

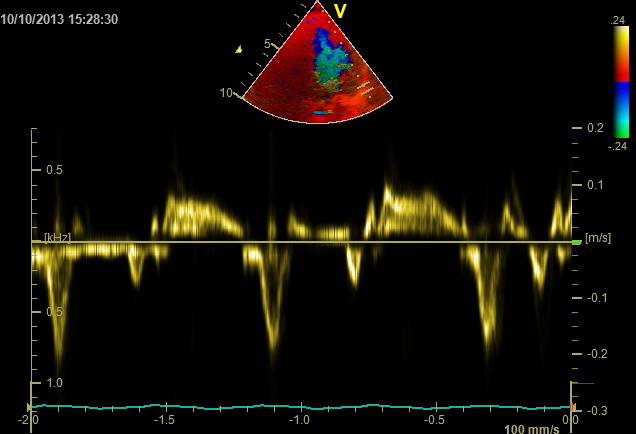

Supplement: S1 File — (ZIP) [file pone.0143744.s002.zip › Image312.jpg]

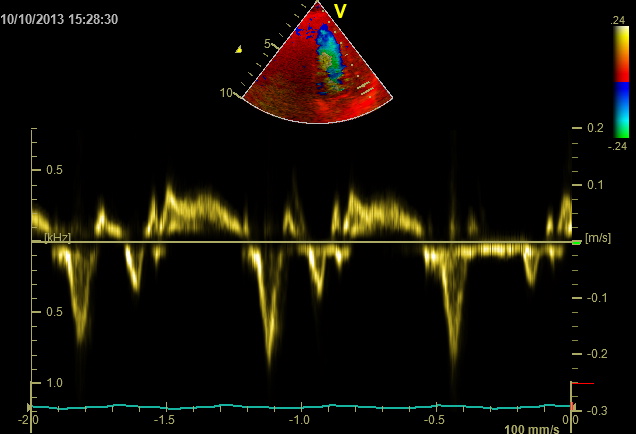

Supplement: S1 File — (ZIP) [file pone.0143744.s002.zip › Image313.jpg]

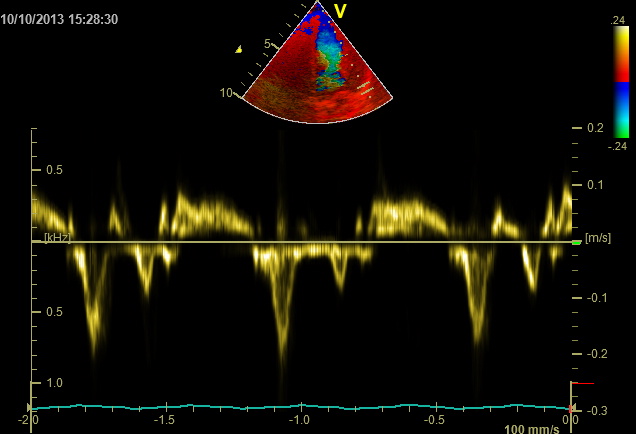

Supplement: S1 File — (ZIP) [file pone.0143744.s002.zip › Image314.jpg]

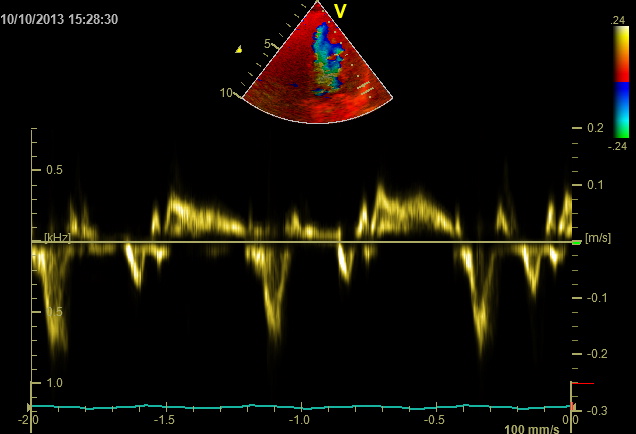

Supplement: S1 File — (ZIP) [file pone.0143744.s002.zip › Image315.jpg]

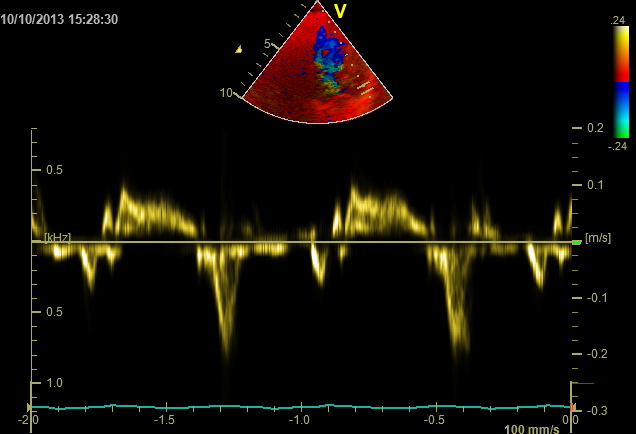

Supplement: S1 File — (ZIP) [file pone.0143744.s002.zip › Image316.jpg]

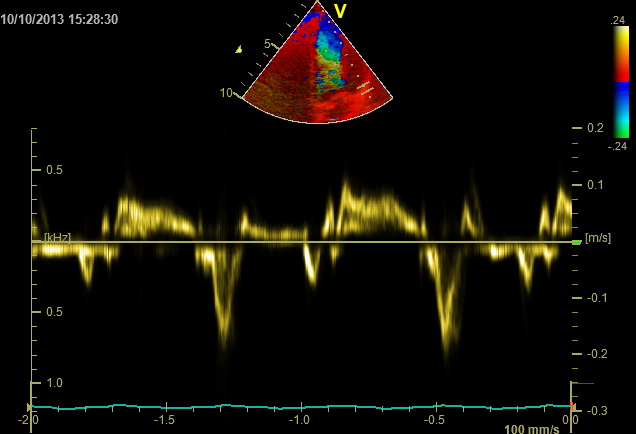

Supplement: S1 File — (ZIP) [file pone.0143744.s002.zip › Image317.jpg]

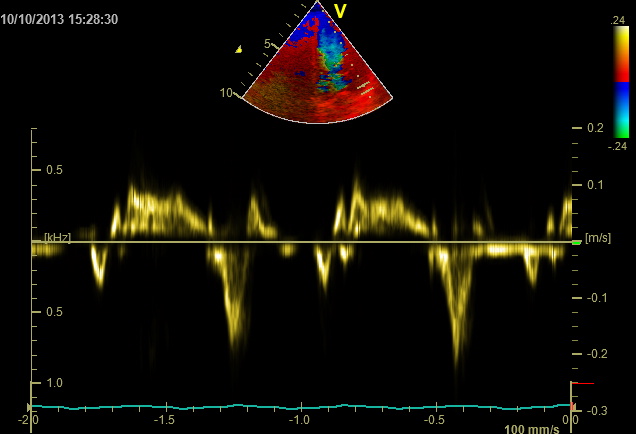

Supplement: S1 File — (ZIP) [file pone.0143744.s002.zip › Image318.jpg]

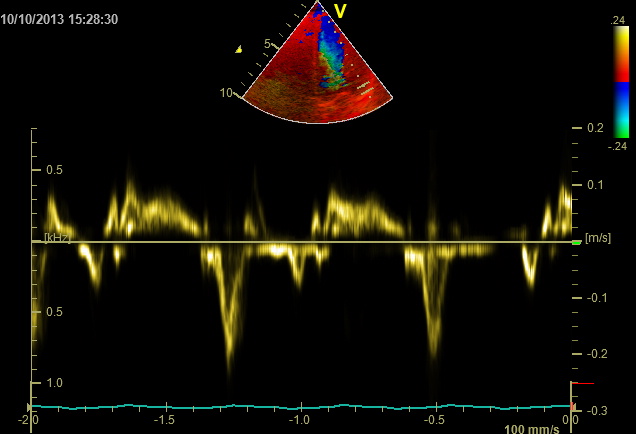

Supplement: S1 File — (ZIP) [file pone.0143744.s002.zip › Image319.jpg]

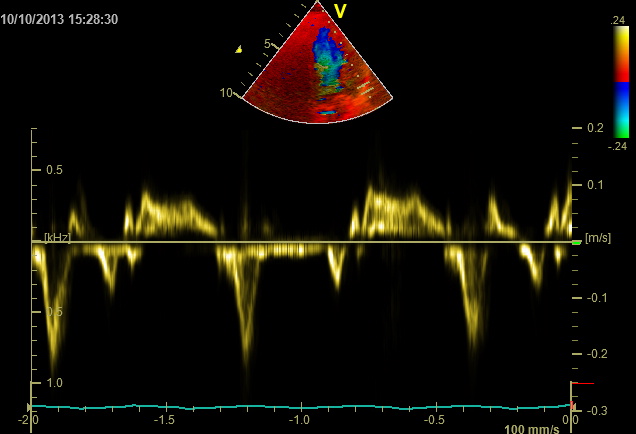

Supplement: S1 File — (ZIP) [file pone.0143744.s002.zip › Image320.jpg]

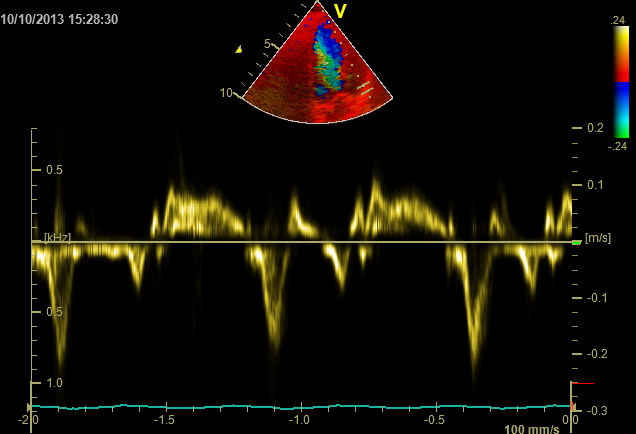

Supplement: S1 File — (ZIP) [file pone.0143744.s002.zip › Image321.jpg]

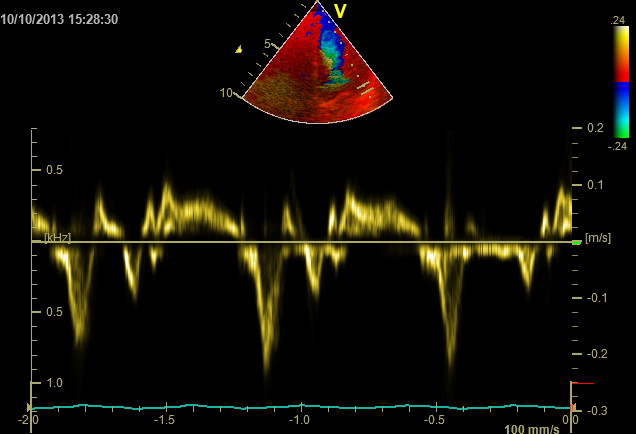

Supplement: S1 File — (ZIP) [file pone.0143744.s002.zip › Image322.jpg]

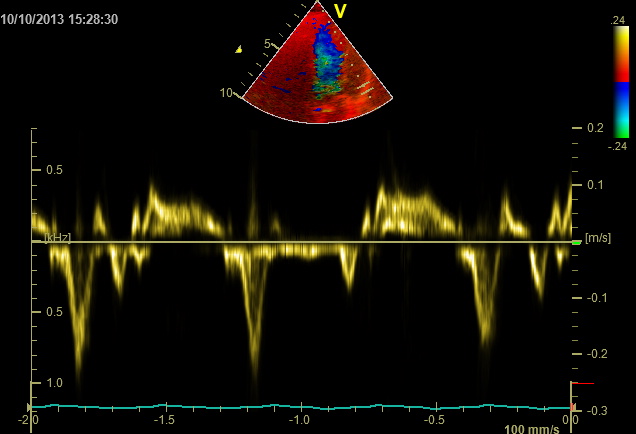

Supplement: S1 File — (ZIP) [file pone.0143744.s002.zip › Image323.jpg]

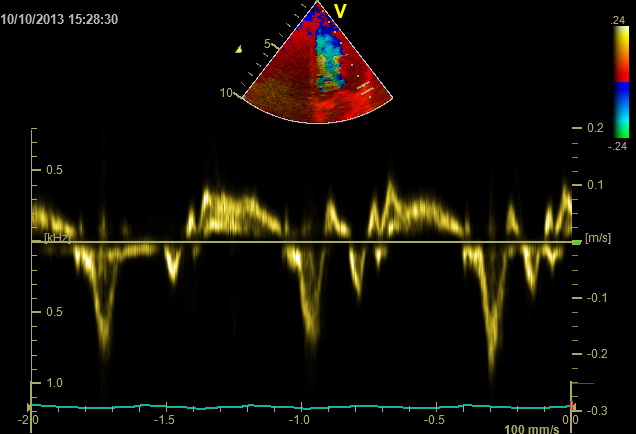

Supplement: S1 File — (ZIP) [file pone.0143744.s002.zip › Image324.jpg]

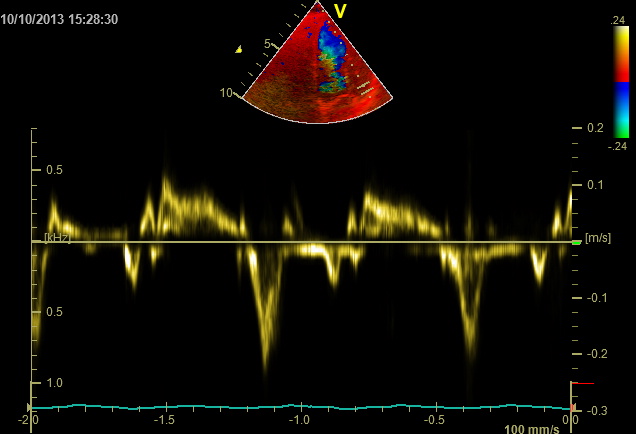

Supplement: S1 File — (ZIP) [file pone.0143744.s002.zip › Image325.jpg]

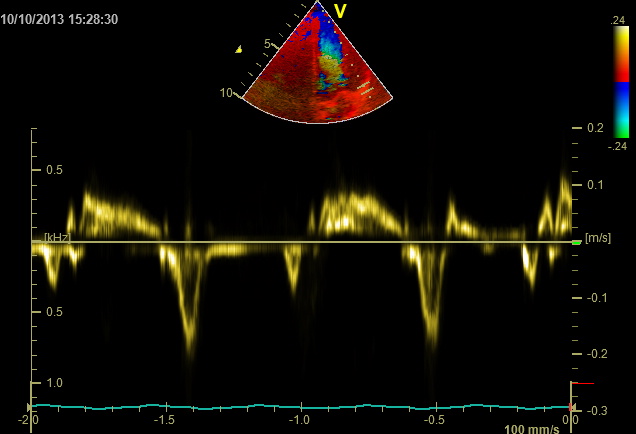

Supplement: S1 File — (ZIP) [file pone.0143744.s002.zip › Image326.jpg]

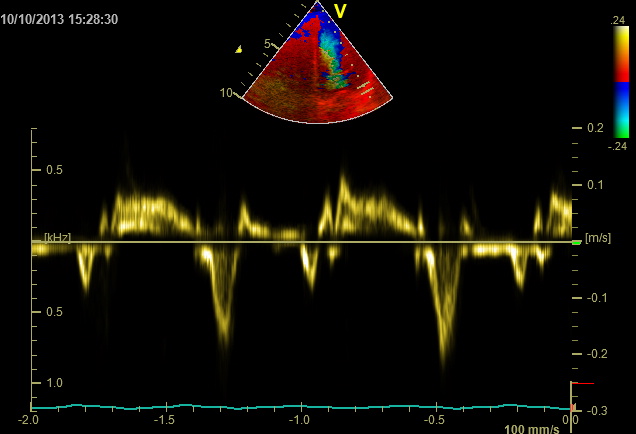

Supplement: S1 File — (ZIP) [file pone.0143744.s002.zip › Image327.jpg]

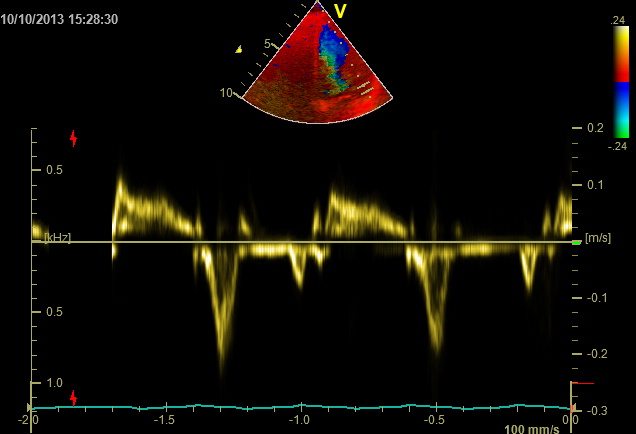

Supplement: S1 File — (ZIP) [file pone.0143744.s002.zip › Image328.jpg]

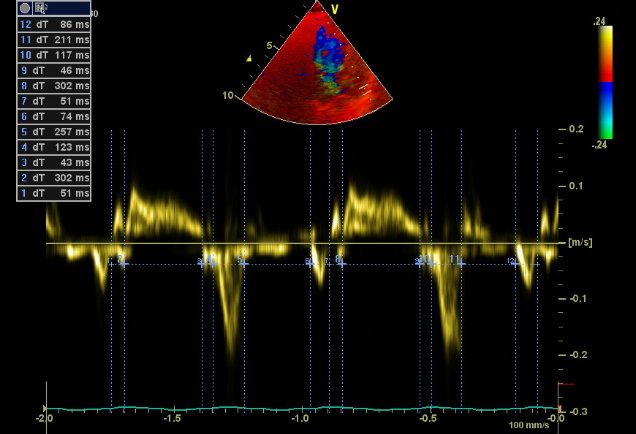

Supplement: S1 File — (ZIP) [file pone.0143744.s002.zip › Image329.jpg]

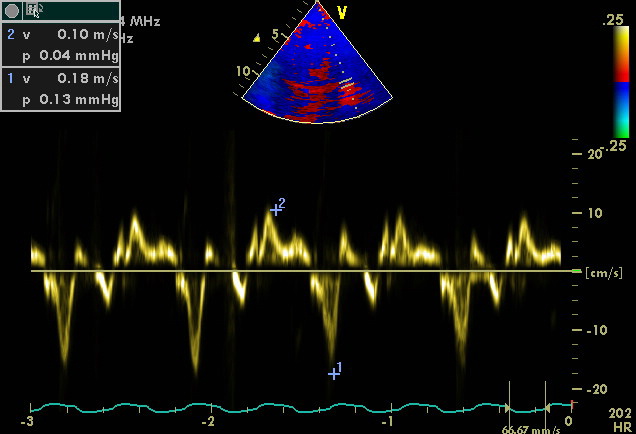

Supplement: S1 File — (ZIP) [file pone.0143744.s002.zip › Image401.jpg]

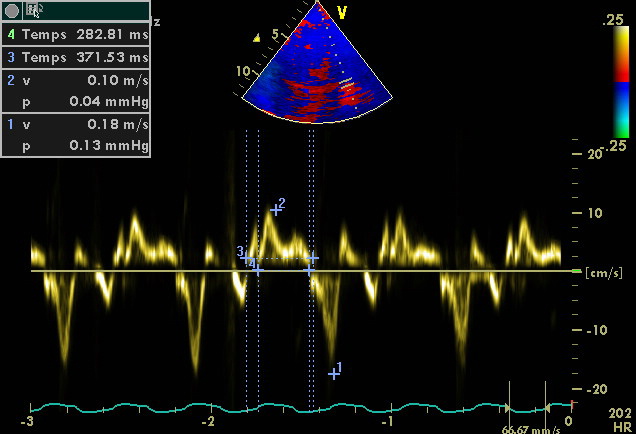

Supplement: S1 File — (ZIP) [file pone.0143744.s002.zip › Image402.jpg]

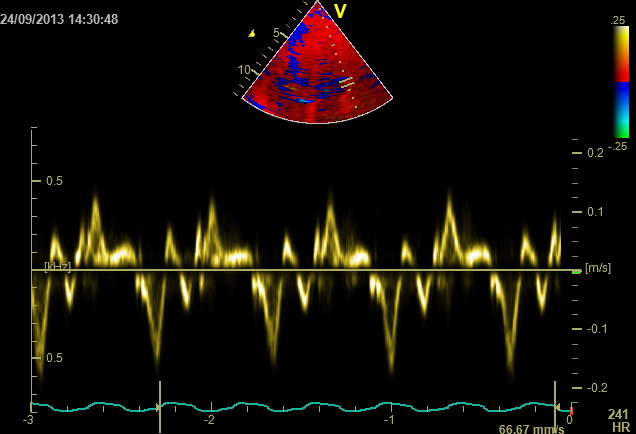

Supplement: S1 File — (ZIP) [file pone.0143744.s002.zip › Image403.jpg]

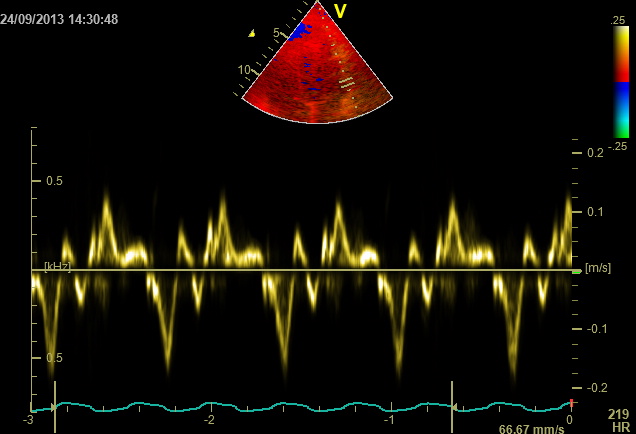

Supplement: S1 File — (ZIP) [file pone.0143744.s002.zip › Image404.jpg]

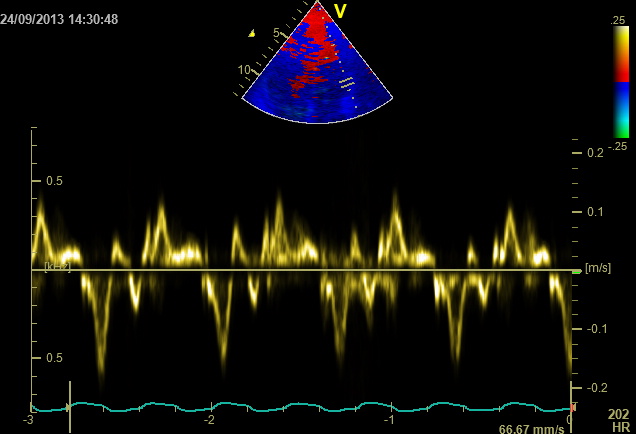

Supplement: S1 File — (ZIP) [file pone.0143744.s002.zip › Image405.jpg]

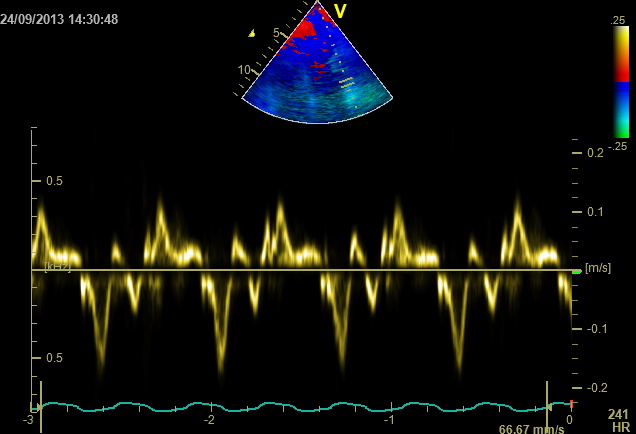

Supplement: S1 File — (ZIP) [file pone.0143744.s002.zip › Image406.jpg]
