# Supplementary figures and images for: Is High Temporal Resolution Achievable for Paediatric Cardiac Acquisitions during Several Heart Beats? Illustration with Cardiac Phase Contrast Cine-MRI (part 2 of 2)
Source: PLoS One. 2015 Nov 24;10(11):e0143744. doi: 10.1371/journal.pone.0143744 (PMC4658039; doi:10.1371/journal.pone.0143744)

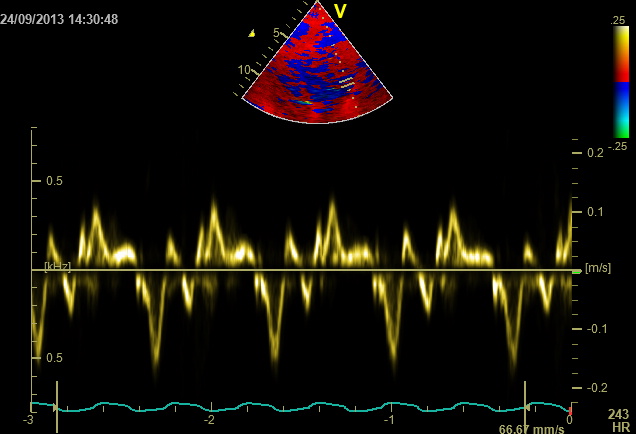

Supplement: S1 File — (ZIP) [file pone.0143744.s002.zip › Image407.jpg]

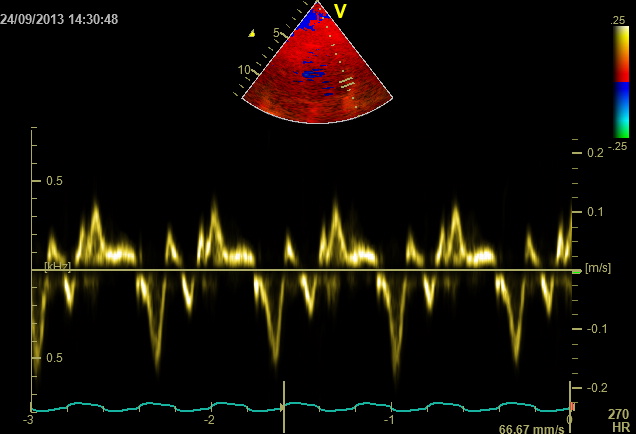

Supplement: S1 File — (ZIP) [file pone.0143744.s002.zip › Image408.jpg]

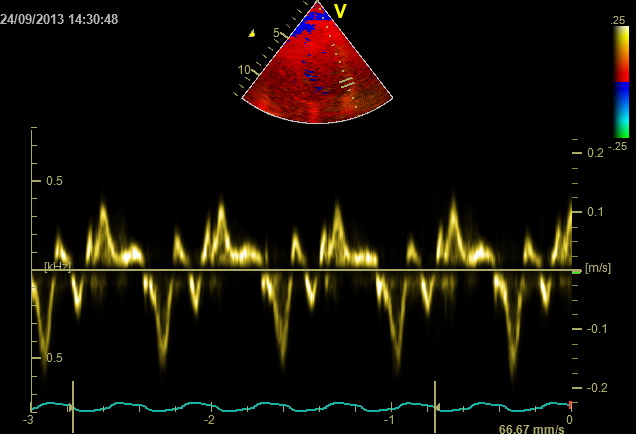

Supplement: S1 File — (ZIP) [file pone.0143744.s002.zip › Image409.jpg]

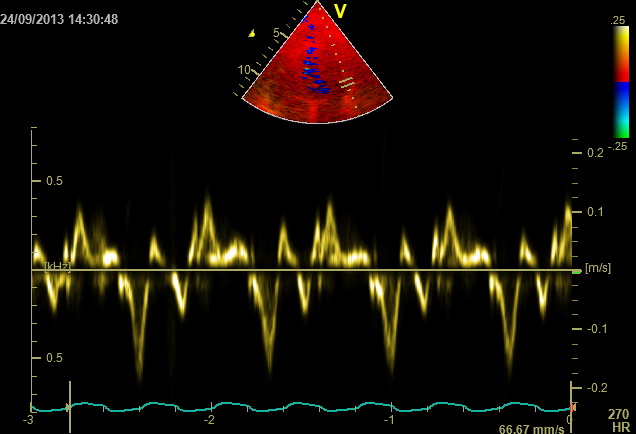

Supplement: S1 File — (ZIP) [file pone.0143744.s002.zip › Image410.jpg]

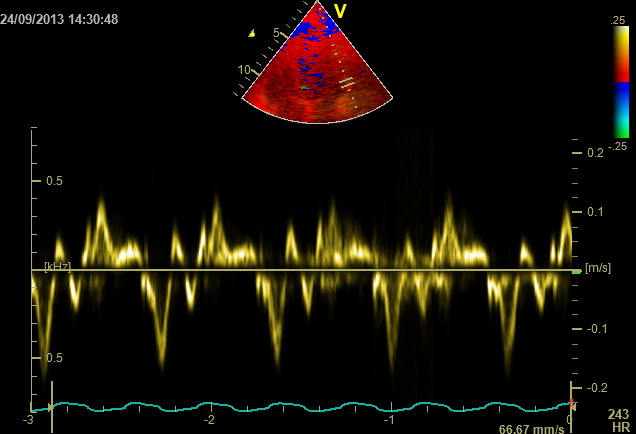

Supplement: S1 File — (ZIP) [file pone.0143744.s002.zip › Image411.jpg]

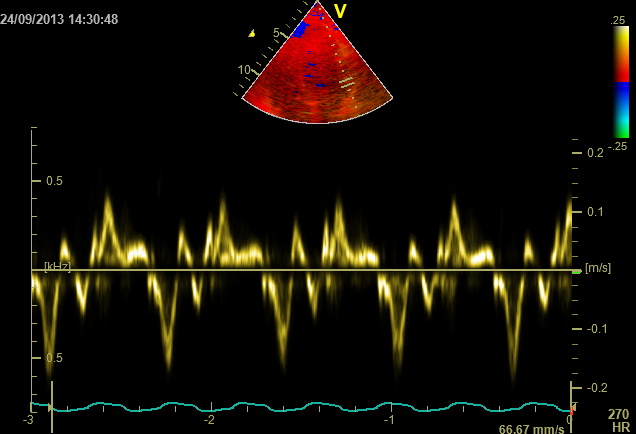

Supplement: S1 File — (ZIP) [file pone.0143744.s002.zip › Image412.jpg]

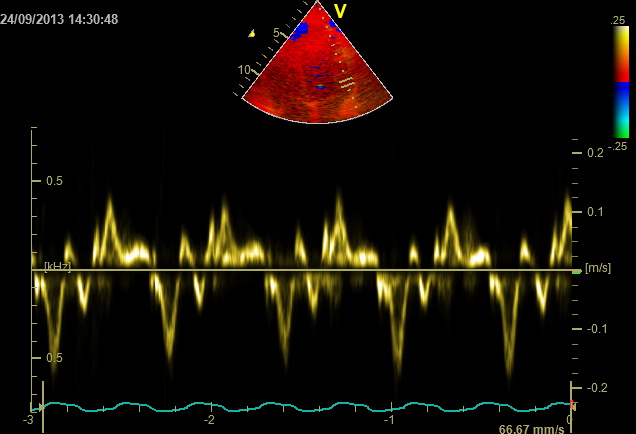

Supplement: S1 File — (ZIP) [file pone.0143744.s002.zip › Image413.jpg]

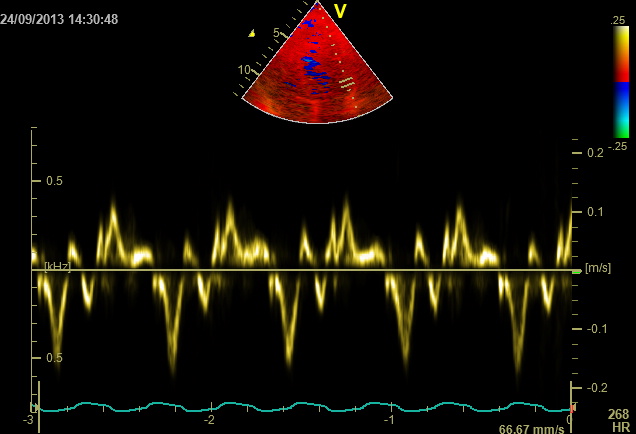

Supplement: S1 File — (ZIP) [file pone.0143744.s002.zip › Image414.jpg]

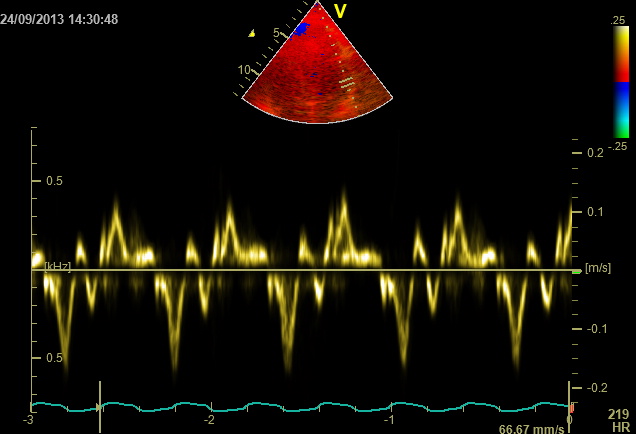

Supplement: S1 File — (ZIP) [file pone.0143744.s002.zip › Image415.jpg]

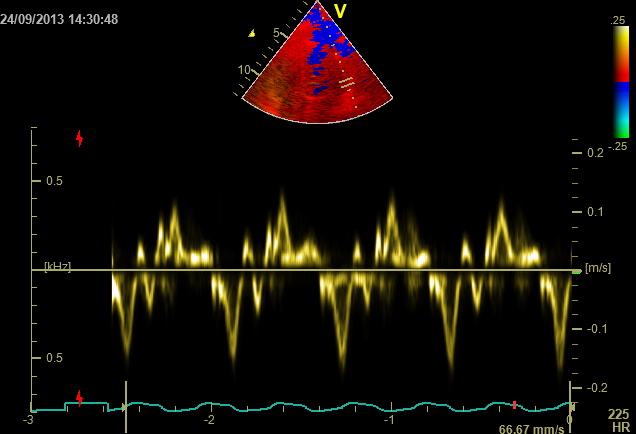

Supplement: S1 File — (ZIP) [file pone.0143744.s002.zip › Image416.jpg]

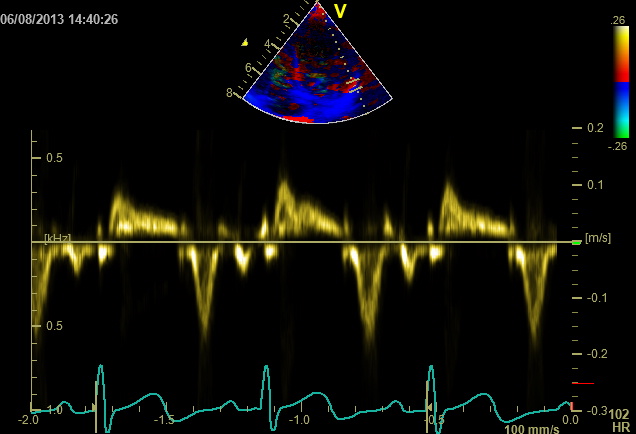

Supplement: S1 File — (ZIP) [file pone.0143744.s002.zip › Image501.jpg]

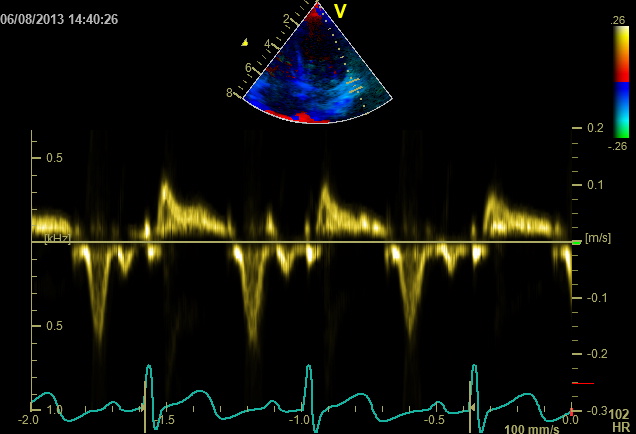

Supplement: S1 File — (ZIP) [file pone.0143744.s002.zip › Image502.jpg]

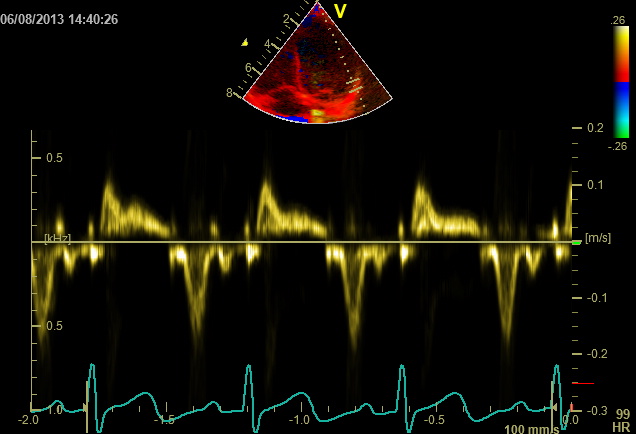

Supplement: S1 File — (ZIP) [file pone.0143744.s002.zip › Image503.jpg]

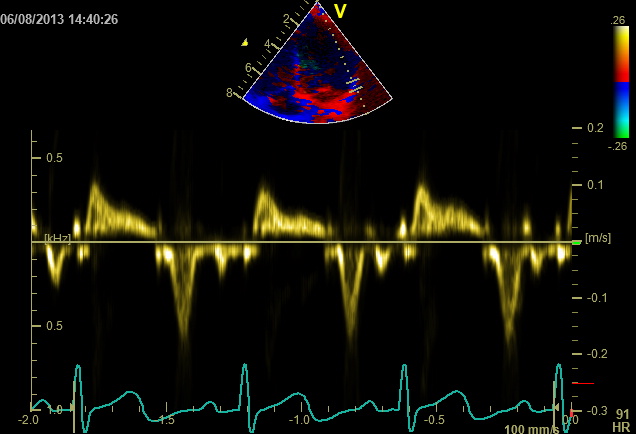

Supplement: S1 File — (ZIP) [file pone.0143744.s002.zip › Image504.jpg]

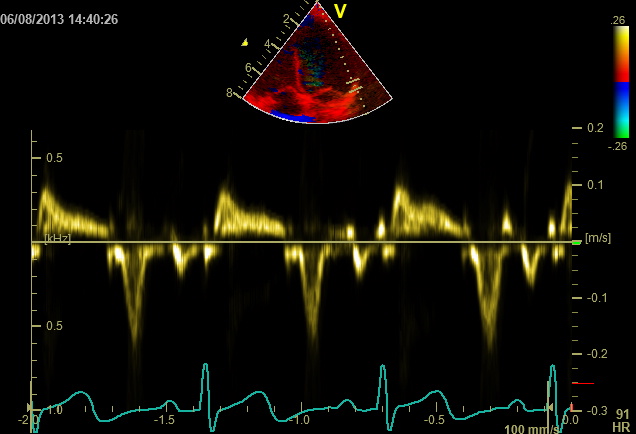

Supplement: S1 File — (ZIP) [file pone.0143744.s002.zip › Image505.jpg]

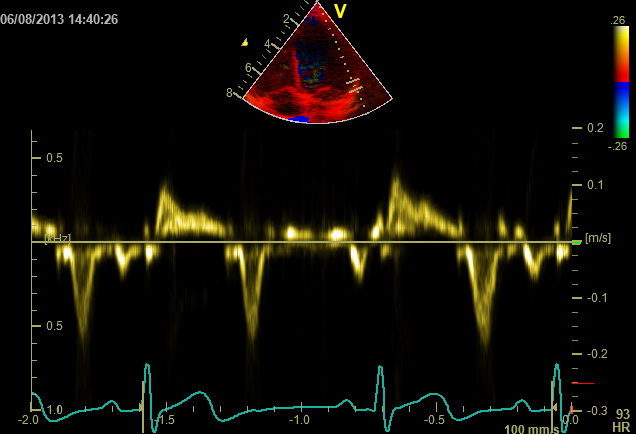

Supplement: S1 File — (ZIP) [file pone.0143744.s002.zip › Image506.jpg]

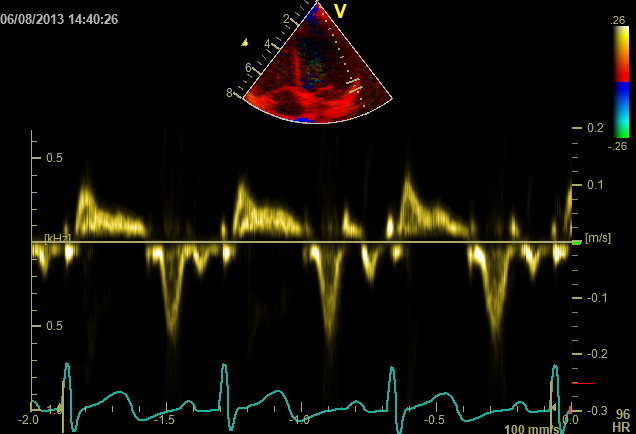

Supplement: S1 File — (ZIP) [file pone.0143744.s002.zip › Image507.jpg]

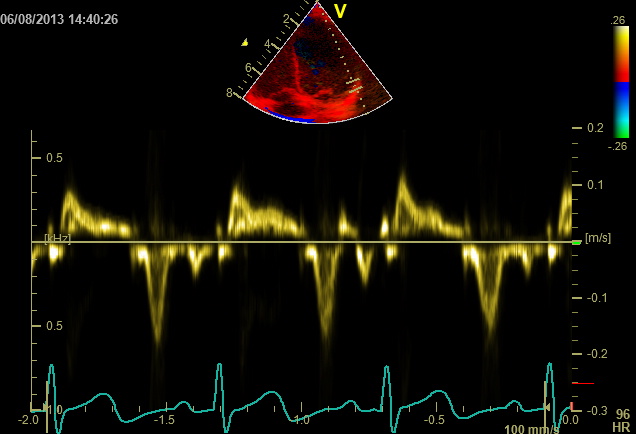

Supplement: S1 File — (ZIP) [file pone.0143744.s002.zip › Image508.jpg]

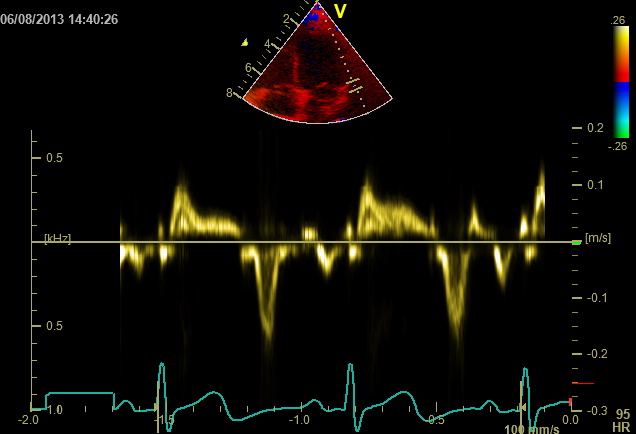

Supplement: S1 File — (ZIP) [file pone.0143744.s002.zip › Image509.jpg]

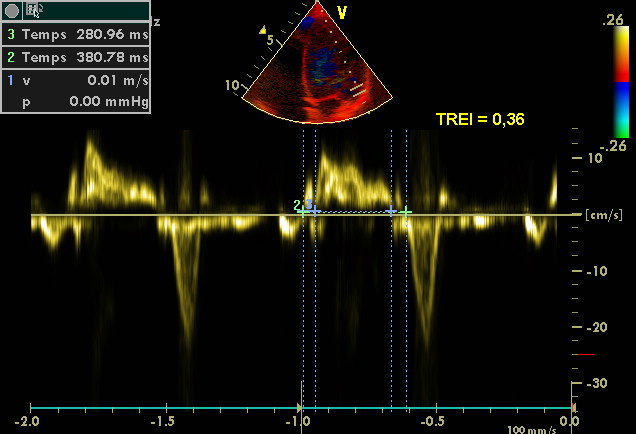

Supplement: S1 File — (ZIP) [file pone.0143744.s002.zip › Image601.jpg]

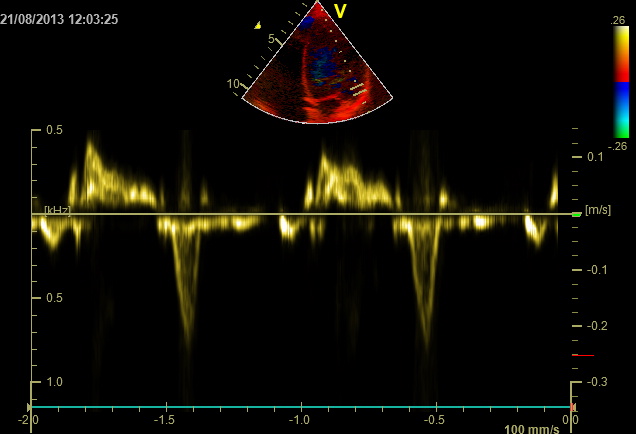

Supplement: S1 File — (ZIP) [file pone.0143744.s002.zip › Image602.jpg]

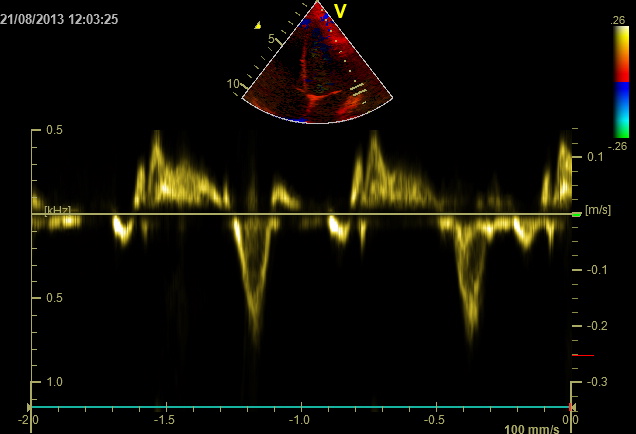

Supplement: S1 File — (ZIP) [file pone.0143744.s002.zip › Image603.jpg]

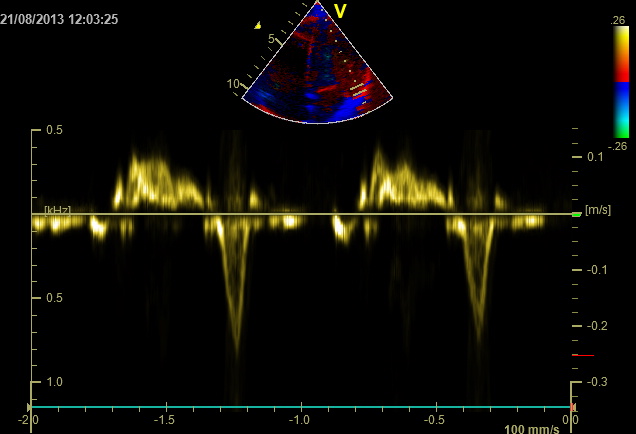

Supplement: S1 File — (ZIP) [file pone.0143744.s002.zip › Image604.jpg]

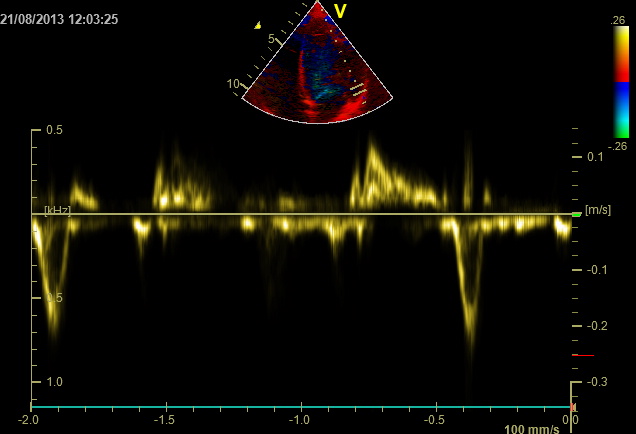

Supplement: S1 File — (ZIP) [file pone.0143744.s002.zip › Image605.jpg]

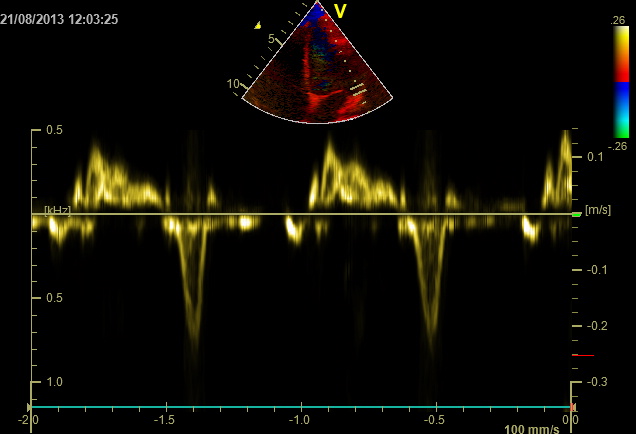

Supplement: S1 File — (ZIP) [file pone.0143744.s002.zip › Image606.jpg]

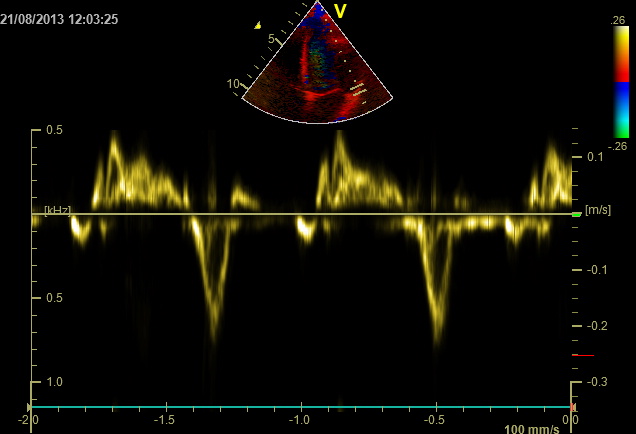

Supplement: S1 File — (ZIP) [file pone.0143744.s002.zip › Image607.jpg]

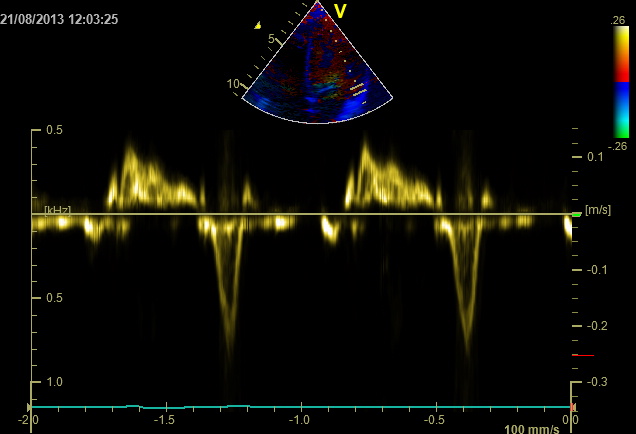

Supplement: S1 File — (ZIP) [file pone.0143744.s002.zip › Image608.jpg]

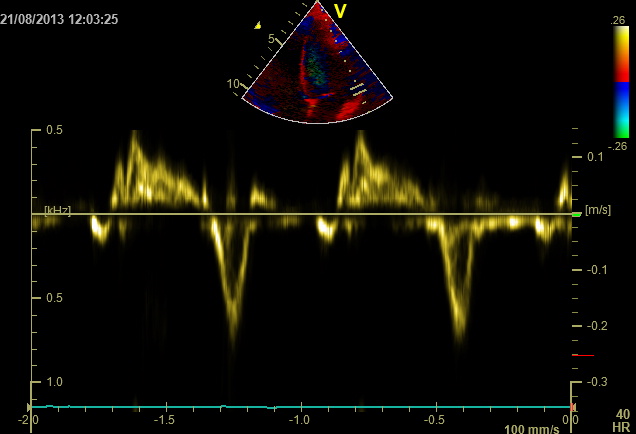

Supplement: S1 File — (ZIP) [file pone.0143744.s002.zip › Image609.jpg]

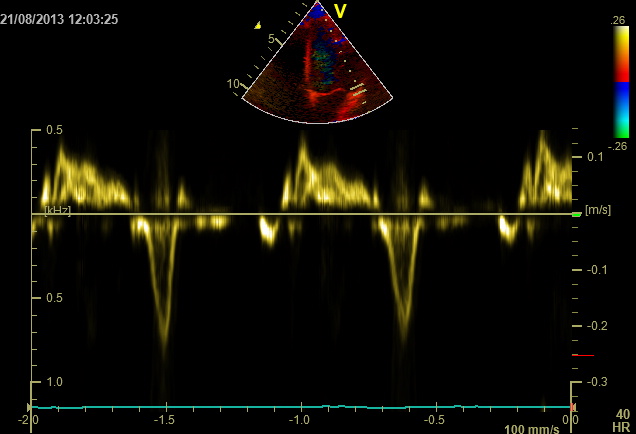

Supplement: S1 File — (ZIP) [file pone.0143744.s002.zip › Image610.jpg]

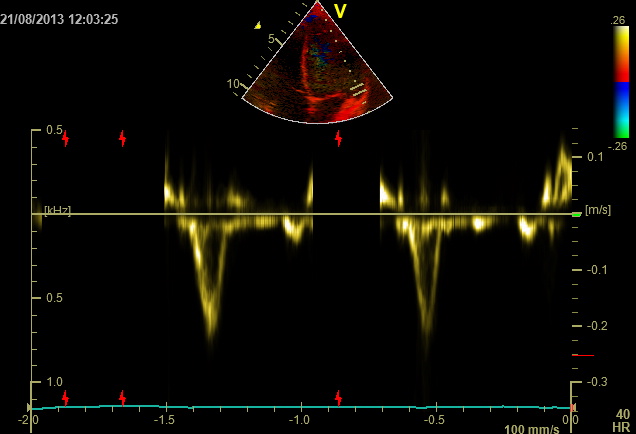

Supplement: S1 File — (ZIP) [file pone.0143744.s002.zip › Image611.jpg]
